# Genome-wide functional analysis reveals that autophagy is necessary for growth, sporulation, deoxynivalenol production and virulence in *Fusarium graminearum*

**DOI:** 10.1038/s41598-017-11640-z

**Published:** 2017-09-11

**Authors:** Wuyun Lv, Chunyan Wang, Nan Yang, Yawei Que, Nicholas J. Talbot, Zhengyi Wang

**Affiliations:** 1 State Key Laboratory for Rice Biology, Institute of Biotechnology, Zhejiang University, Hangzhou, 310029 China; 2 School of Biosciences, University of Exeter, Geoffrey Pope Building, Exeter, EX4 4QD United Kingdom

## Abstract

Autophagy is a conserved cellular recycling and trafficking pathway in eukaryotic cells and has been reported to be important in the virulence of a number of microbial pathogens. Here, we report genome-wide identification and characterization of autophagy-related genes (*ATGs*) in the wheat pathogenic fungus *Fusarium graminearum*. We identified twenty-eight genes associated with the regulation and operation of autophagy in *F. graminearum*. Using targeted gene deletion, we generated a set of 28 isogenic mutants. Autophagy mutants were classified into two groups by differences in their growth patterns. Radial growth of 18 Group 1 *ATG* mutants was significantly reduced compared to the wild-type strain PH-1, while 10 Group 2 mutants grew normally. Loss of any of the *ATG* genes, except *FgATG17*, prevented the fungus from causing Fusarium head blight disease. Moreover, subsets of autophagy genes were necessary for asexual/sexual differentiation and deoxynivalenol (DON) production, respectively. *FgATG1* and *FgATG5* were investigated in detail and showed severe defects in autophagy. Taken together, we conclude that autophagy plays a critical role in growth, asexual/sexual sporulation, deoxynivalenol production and virulence in *F. graminearum*.

## Introduction

*Fusarium graminearum* Schwabe (teleomorph *Gibberella zeae* (Schweinitz) Petch) is a homothallic filamentous ascomycete fungus and the causal agent of Fusarium head blight (FHB) or head scab disease of wheat, barley, rice and other small grain cereals worldwide^1–3^. Damage from head scab results in reduced yield, discolored, shrived “tombstone” kernels, contamination with mycotoxins, and reduction in seed quality^4^. *F. graminearum* produces Trichothecene mycotoxins, such as nivalenol (NIV) and deoxynivalenol (DON) and an estrogenic mycotoxin, zearalenone (ZEN). Contamination of cereals and feeds with these mycotoxins sporadically causes food and feed-borne intoxication in man and farm animals^5, 6^. FHB is one of the most economically important diseases of grain cereals^3^ and is not controlled well by any current strategies. It is therefore important to understand the infection mechanisms of *F. graminearum* to guide development of more durable control strategies against FHB.

The term “autophagy” was first used by Christian de Duve in 1963 on the occasion of the *Ciba Foundation Symposium on Lysosomes* ^7^. Autophagy is required for maintaining the homeostasis of eukaryotic cells and plays an important role in normal development and differentiation^8^. To date, 38 *ATG* genes (AuTophagy-related Genes) have been identified in *Saccharomyces cerevisiae*, and the biological properties of most of the corresponding Atg proteins have now been characterized^9, 10^. Autophagy can be divided into three main types based on recognized mechanisms and functions– macroautophagy, microautophagy, and chaperone-mediated autophagy^11, 12^. Macroautophagy is generally referred to simply as autophagy, and is the most well characterized process of the three processes, used for sequestration and degradation of cytosolic components in a process that uses specialized cytosolic double membrane vesicles called autophagosomes, which ultimately fuse with the lysosome/vacuole, releasing the contents of the vesicle and subsequently breaking down, as proteolysis occurs^8, 12–14^. Chaperone-mediated autophagy degrades soluble proteins that contain a motif biochemically related to the pentapeptide KFERQ^11, 15^. Although autophagy can be nonspecific, there are many selective forms of autophagy in *S. cerevisiae*, including the cytoplasm-to-vacuole (Cvt) targeting pathway, mitophagy, pexophagy, and other specific forms that target specific organelles. In filamentous fungi, autophagy has been shown to be involved in vegetative growth, asexual/sexual differentiation, environmental stresses, and virulence^16–22^. In the rice blast fungus *Magnaporthe oryzae*, a set of 22 isogenic mutants differing by a single component of the predicted autophagic machinery of the fungus showed that autophagy is necessary for rice blast disease^17^. The *Mgatg8* mutant impaired in autophagy arrests conidial cell death and this renders *M. grisea* non-pathogenic^23^. Liu *et al*. have independently shown that the autophagy genes, *MgATG1*, *MgATG4*, *MgATG5*, *MgATG8* and *MgATG9* are required for pathogenesis in *M. oryzae* ^18, 24–26^. In the corn smut fungus *Ustilago maydis*, autophagy is also involved in pathogenicity^27^. Recently, Yanagisawa and colleagues have analyzed the function of *Aoatg1* and detected the Cvt pathway in *Aspergillus oryzae* ^28^. More recently, in the endophytic fungus *Harpophora oryzae*, it has been shown that autophagy is required for vegetative growth, sporulation and virulence^29^. Selective autophagy may also be significant in pathogenesis, because it has been shown that Atg26-mediated pexophagy is necessary for appressorium-mediated plant infection in the hemi-biotrophic plant pathogenic fungus *Colletotrichum orbiculare* ^16^. Similarly, He *et al*. revealed that Atg24-assisted mitophagy in foot cells is necessary for proper asexual differentiation and efficient conidiogenesis in *M. oryzae* ^30^.

Recently, two autophagy-related genes, *FgATG15* and *FgATG8*, have been functionally characterized in *F. graminearum* ^20, 31^. *FgATG15* is involved in fungal growth, aerial hyphae production, conidia production and germination and important for lipid turnover and plant infection^31^. *FgATG8* is related to linear growth rate, formation of aerial mycelium, use of storage lipid droplets, growth over an inert plastic surface, infection and formation of reproductive structures^20^. To further understand the biological roles of autophagy in morphogenesis and plant infection, we identified all 26 *ATG* genes, except the previously reported *FgATG8* and *FgATG15*, in the genome of *F. graminearum*. We generated targeted deletion mutants of 28 *ATG* genes and demonstrated that loss of any of the *ATGs*, except *FgATG17*, prevents the fungus from causing head blight disease. Moreover, we observed that autophagy is important for vegetative growth, asexual/sexual differentiation and DON production. We conclude that autophagy plays a critical role in growth, sporulation, deoxynivalenol production and virulence in *F. graminearum*.

## Results

### Identification of *ATG* genes in *F. graminearum*

We first carried out a genome-wide search for Atg protein-encoding genes in the *F. graminearum* genome database using *S. cerevisiae* functional annotations as a guide and, in this way, we defined a set of 28 *ATG* genes, which are described in detail in Table S1. Non-selective macroautophagy, often referred to simply as autophagy, is a dynamic process^8^, but can be conceptually divided into several steps, based on studies in yeast. According to molecular analysis of a battery of autophagy-related genes^32–38^, the predicted genes involved in autophagy of *F. graminearum* could be functionally separated into those that putatively play a role in the induction of autophagy (*FgATG1*, *FgATG13* and *FgATG17*), vesicle nucleation (*FgATG18*, *FgATG20*, *FgATG24* and *FgATG29*), autophagosome expansion *(FgATG3*, *FgATG4*, *FgATG5*, *FgATG7*, *FgATG8*, *FgATG10*, *FgATG12* and *FgATG16*), docking and fusion, and recycling (*FgATG2*, *FgATG9*, *FgATG15*, *FgATG18* and *FgATG22*). By reference to Atg proteins specific for selective autophagy in yeast^39, 40^, *FgATG11*, *FgATG20*, *FgATG23*, *FgATG24*, *FgATG26*, *FgATG27*, *FgATG28*, *FgATG33* and *FgATG37* may be required for mitophagy, pexophagy, or the Cvt pathway in *F. graminearum*. However, homologs of yeast *ATG19*, *ATG21*, *ATG25*, *ATG30*, *ATG31*, *ATG32*, *ATG34*, *ATG36*, *ATG38*, *ATG39*, *ATG40* and *ATG41* were not found in *F. graminearum* (Table S1).

### Autophagy is required for proper vegetative growth in *F. graminearum*

**Figure 1:**
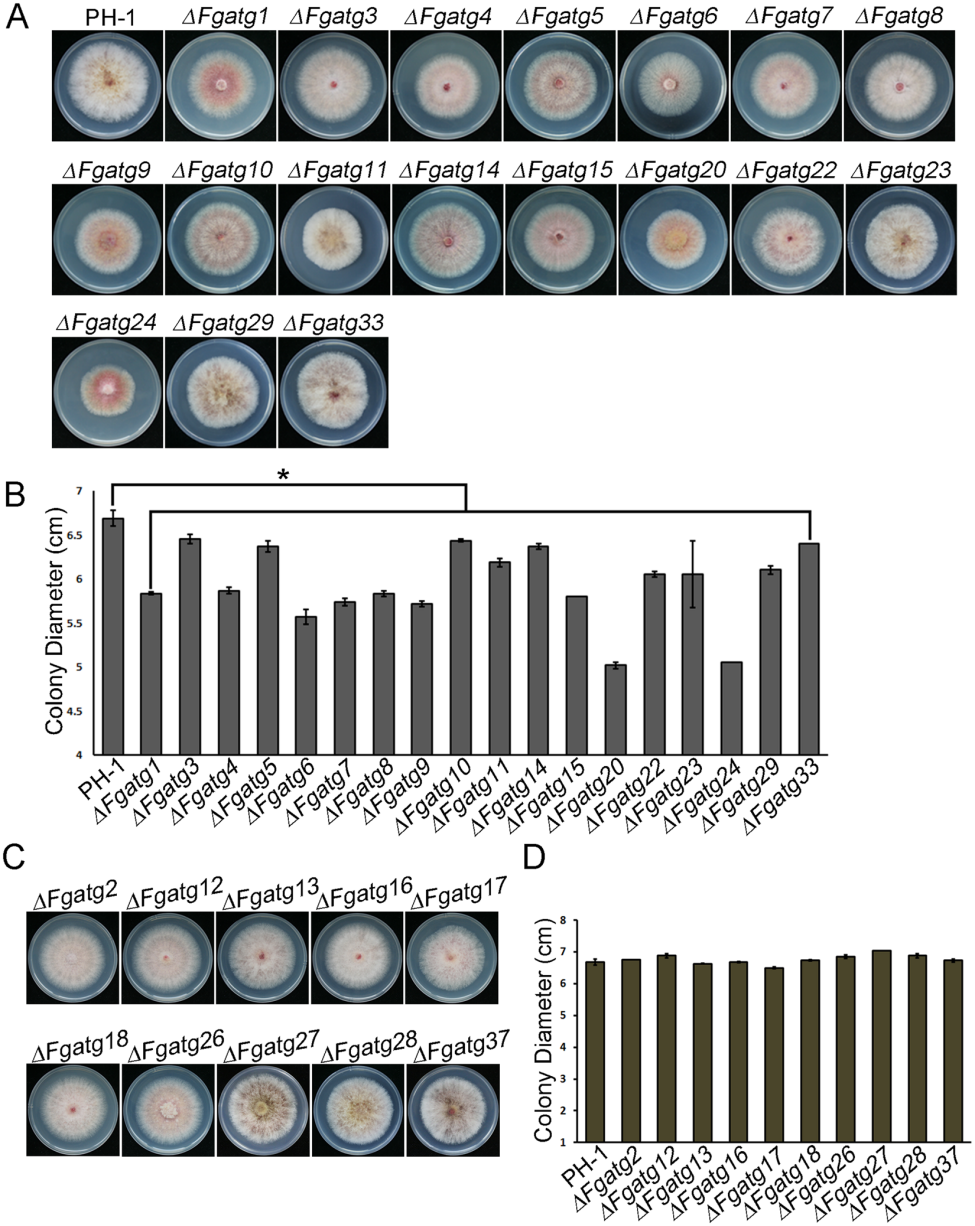
Defects of *ATG* mutants in hyphal growth. (**A**) Colonies of the Group 1 mutants. (**B**) Bar chart showing colony diameters of PH-1 and the Group 1 mutants. (**C**) Colonies of the Group 2 mutants. (**D**) Bar chart showing colony diameters of PH-1 and the Group 2 mutants. The wild-type strain PH-1 and *ATG* mutants were grown on PDA plates. Photographs were taken after incubation on PDA plates at 25 °C for 3 days. Linear bars in each column denote standard errors of three experiments. An asterisk indicates significant difference of colony diameter (*P* < 0.05).

To determine the function of the putative *ATG* gene*s* in *F. graminearum*, we generated targeted gene deletion mutants of 28 *ATGs*, which were confirmed by PCR and Southern blot. Confirmation of four mutants, *∆Fgatg1*, *∆Fgatg5*, *∆Fgatg20* and *∆Fgatg24*, by Southern blot analysis is shown in Fig. S1. Based on differences in growing patterns, the 28 *ATG* mutants could be divided into two groups. In Group 1, colonies showed a statistically significant difference from the wild-type strain PH-1 in radial growth under nutrient-rich conditions (PDA plates). These included *∆Fgatg1*, *∆Fgatg3*, *∆Fgatg4*, *∆Fgatg5*, *∆Fgatg6*, *∆Fgatg7*, *∆Fgatg8*, *∆Fgatg9*, *∆Fgatg10*, *∆Fgatg11*, *∆Fgatg14*, *∆Fgatg15*, *∆Fgatg20*, *∆Fgatg22*, *∆Fgatg23*, *∆Fgatg24*, *∆Fgatg29* and *∆Fgatg33* (Fig. 1A,B). For example, colony diameters of the *∆Fgatg1* and *∆Fgatg20* mutants were (5.83 ± 0.02) cm and (5.02 ± 0.03) cm after incubation for 3 days on PDA at 25 °C, respectively, which was significantly smaller than (6.68 ± 0.09) cm of the PH-1 strain. In Group 2 mutants, no significant difference in growth rate was observed compared to the wild-type strain. These mutants included *∆Fgatg2*, *∆Fgatg12*, *∆Fgatg13*, *∆Fgatg16*, *∆Fgatg17*, *∆Fgatg18*, *∆Fgatg26*, *∆Fgatg27*, *∆Fgatg28* and *∆Fgatg37* (Fig. 1C,D). On PDA plates, colonies of the wild-type strain PH-1 produced dense aerial mycelium, while colonies of most *ATG* mutants in the two groups (except *∆Fgatg11*, *∆Fgatg23*, *∆Fgatg27*, *∆Fgatg28*, *∆Fgatg29*, *∆Fgatg33* and *∆Fgatg37*) showed significantly decreased development of aerial mycelium compared to PH-1 (Fig. 1A,C). These results indicate that autophagy is necessary for proper vegetative growth in *F. graminearum*.

### Autophagy plays a critical role in asexual/sexual reproduction in *F. graminearum*

**Figure 2:**
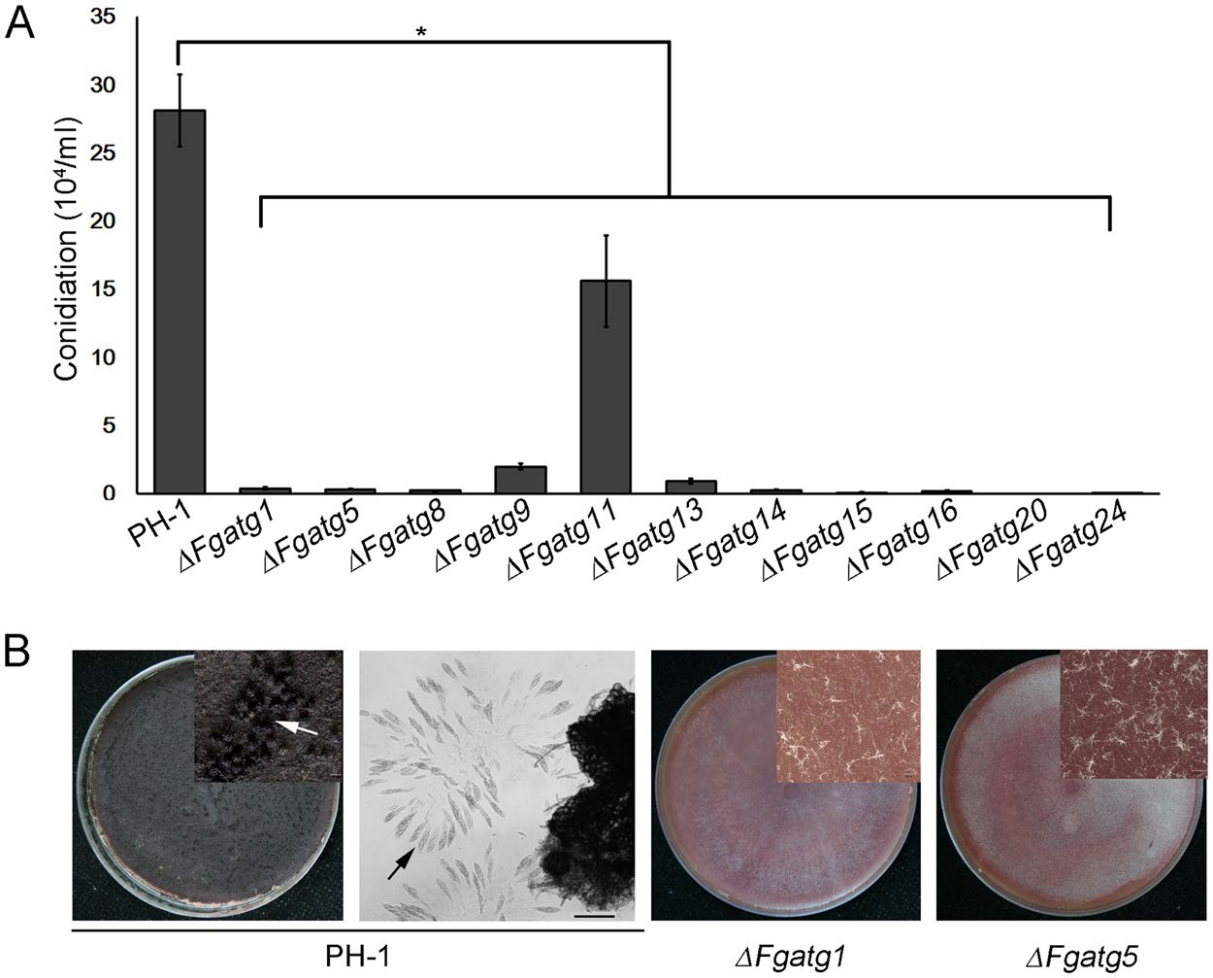
Defects of the *ATG* mutants in asexual/sexual sporulation. (**A**) Bar chart showing the conidial production of indicated strains. Conidia of each strain were harvested from the 1% MBL cultures after incubation at 25 °C for 4 days in a 180 rpm shaker. All tested *ATG* mutants produced less conidia than PH-1. Linear bars in each column denote standard errors of three repeats. Asterisks indicate significant difference of conidiation (an asterisk, *P* < 0.05). (**B**) Self-crossing plates of the wild-type strain, *∆Fgatg1* and *∆Fgatg5* mutants. The photographs were taken after sexual induction for 14 days. The enlarged images were taken using anatomical lens. Perithecia were only produced by the wild-type PH-1. The asci and ascospores were observed under optical microscope. Arrows indicate perithecia, asci and ascospores. Scale bar = 20 µm.

We next examined conidiogenesis of the wild-type strain and 11 *ATG* mutants by growing cultures in MBL liquid medium. The PH-1 strain typically produced (28.10 ± 2.64) × 10^4^ macroconidia per milliliter from such cultures. Conidiation of *∆Fgatg1*, *∆Fgatg5*, *∆Fgatg8*, *∆Fgatg9*, *∆Fgatg11*, *∆Fgatg13*, *∆Fgatg14*, *∆Fgatg15*, *∆Fgatg16*, *∆Fgatg20* and *∆Fgatg24* was significantly reduced (Fig. 2A). The *∆Fgatg15*, *∆Fgatg20* and *∆Fgatg24* mutants cultured for 4 days in the 1% MBL medium produced hardly any macroconidia. The *∆Fgatg11* mutant produced (15.59 ± 3.35) × 10^4^ macroconidia per milliliter, a decrease of 44.52% compared to PH-1. Conidiation of *∆Fgatg1*, *∆Fgatg5*, *∆Fgatg8*, *∆Fgatg9*, *∆Fgatg13*, *∆Fgatg14* and *∆Fgatg16* was only1.25%, 1.10%, 0.68%, 7.01%, 3.17%, 0.85% and 0.53% that of the wild-type strain, respectively.

Sexual reproduction plays a critical role in FHB epidemic and disease cycle, thus we determined the sexual development of the wild-type strain and two *ATG* mutants on self-mating carrot agar cultures. The wild-type strain PH-1 produced abundant perithecia after two-week self-fertilization. By contrast, *∆Fgatg1* and *∆Fgatg5* mutants completely failed to form any perithecia under the same culture conditions (Fig. 2B). These results suggest that autophagy is important for asexual/sexual sporulation in *F. graminearum*.

### Autophagy is required for full virulence in *F. graminearum*

**Figure 3:**
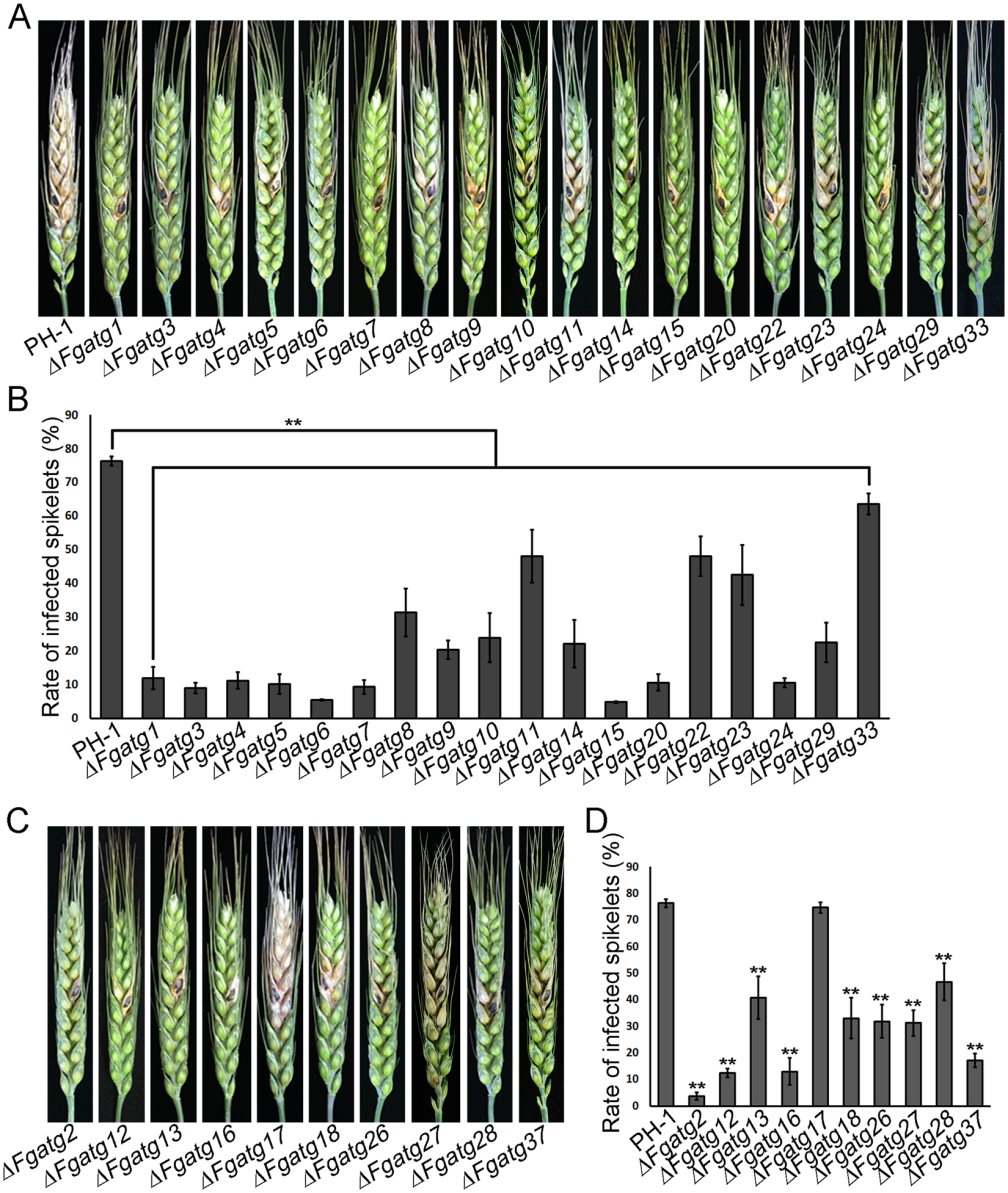
Pathogenicity assays of the *ATG* mutants in *F. graminearum*. (**A**) Virulence of PH-1 and the Group 1 mutants was determined on wheat heads. (**B**) Bar chart showing the rate of infected spikelets of Group 1 mutants. (**C**) Virulence of PH-1 and the Group 2 mutants was determined. (**D**) Bar chart showing the rate of infected spikelets of Group 2 mutants. Wheat heads were point-inoculated with mycelial plugs from PH-1 and the mutants. Infected wheat heads and rate of infected spikelets were determined at 14 days after inoculation. Linear bars in each column denote standard errors of ten repeats. Two asterisks indicate significant difference of infected spikelets (*P* < 0.01).

Virulence assays were performed by point inoculation of flowering wheat heads with mycelial plugs from the wild-type PH-1 and *ATG* mutants. At 14 days post-inoculation (dpi), PH-1 caused typical scab symptom on inoculated and nearby spikelets. The rate of infected spikelets inoculated with most *ATG* mutants was significantly reduced compared to PH-1 (*P* < 0.01), while *FgATG17* in Group 2 was fully virulent (Fig. 3). Atg17, as a component of the Atg1 complex, is important for induction of autophagy by starvation in *S. cerevisiae* ^41^. Therefore, we determined the role of *FgATG17* in autophagy and DON production in *F. graminearum*. We found that GFP-FgAtg8 proteolysis was not blocked in the *∆Fgatg17* mutant after induction of autophagy in MM-N liquid medium with 2 mM PMSF for 4 h (Fig. S4A). To determine whether DON production in the *∆Fgatg17* mutant was impaired, an enzyme-linked immunosorbent assay (ELISA) was performed. The results showed that DON production between PH-1 and the *∆Fgatg17* mutant had no significant difference (Fig. S4B). These data suggested that *FgATG17* is not essential for autophagy involved in FgAtg8 and DON production in *F. graminearum*. Furthermore, we observed that *∆Fgatg1*, *∆Fgatg3*, *∆Fgatg6*, *∆Fgatg7*, *∆Fgatg14*, *∆Fgatg15*, *∆Fgatg20*, *∆Fgatg24* in Group 1 and *∆Fgatg2*, *∆Fgatg12*, *∆Fgatg13*, *∆Fgatg16* in Group 2 only caused mild infection in point-inoculated spikelets but did not spread to nearby spikelets (Fig. 3A,C). Although scab symptoms developed in nearby spikelets inoculated with the other *ATG* mutants, the percentage of diseased spikelets was dramatically decreased by contrast to PH-1 (Fig. 3B,D). In addition, the phenotypic defects of *∆Fgatg1*, *∆Fgatg7*, *∆Fgatg13* and *∆Fgatg20*, such as growth, conidiation and virulence, could be fully complemented by re-introduction of the corresponding *ATG* genes, respectively. For instance, in Fig. S3, the defects in vegetative growth of *∆Fgatg1*, *∆Fgatg7*, *∆Fgatg13* and *∆Fgatg20* could be complemented by re-introduction of *FgATG1*, *FgATG7*, *FgATG1*3 and *FgATG20* respectively (Fig. S3). These results indicate that autophagy is required for full virulence to wheat by *F. graminearum*.

### Autophagy is involved in DON biosynthesis in *F. graminearum*

**Figure 4:**
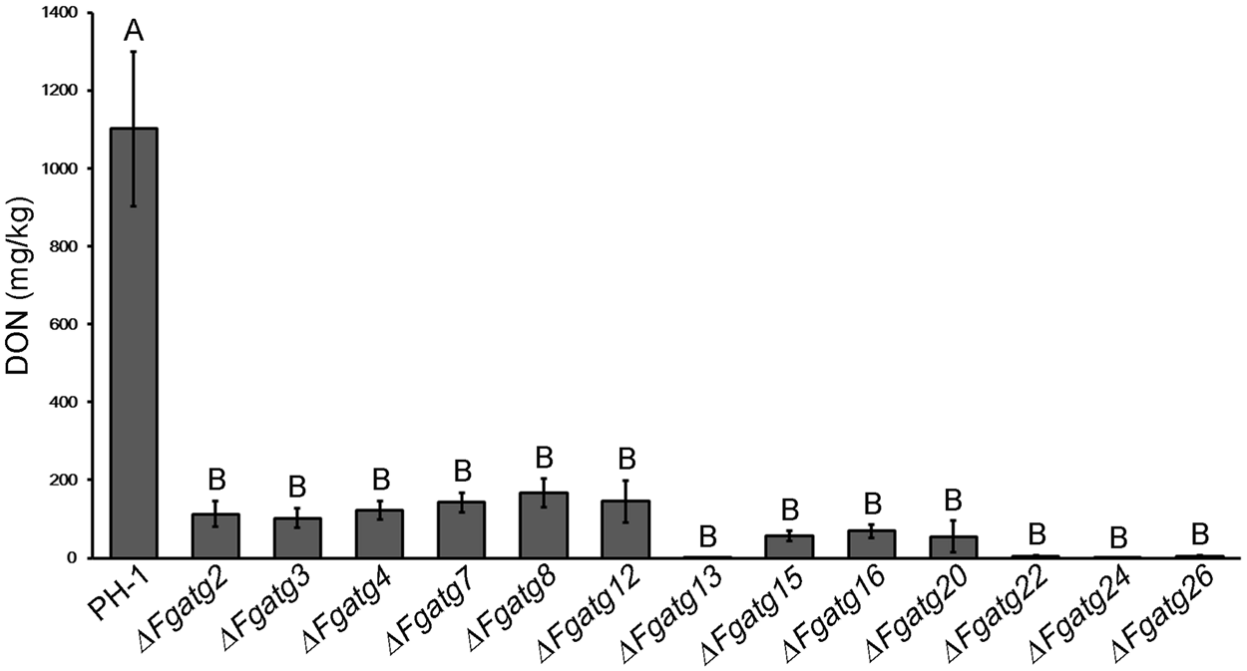
The *ATG* mutants were involved in DON production in *F. graminearum*. Levels of DON in PH-1 and 13 *ATG* mutants were detected in infected wheat kernels at 25 days post-inoculation (dpi). All the tested mutants produced significantly less DON than PH-1. Linear bars in each column represent standard errors of four repeats. Different capital letters indicate a significant different DON level (*P* < 0.01).

Deoxynivalenol (DON) is known to be an important virulence determinant in *F. graminearum* ^42, 43^. Therefore DON production was measured in wheat kernels infected by PH-1 and several *ATG* mutants. The wild-type strain PH-1 produced (1102.04 ± 198.54) milligrams DON per kilogram inoculated wheat kernels. The levels of DON production in the all tested mutants, including *∆Fgatg2*, *∆Fgatg3*, *∆Fgatg4*, *∆Fgatg7*, *∆Fgatg8*, *∆Fgatg12*, *∆Fgatg13*, *∆Fgatg15*, *∆Fgatg16*, *∆Fgatg20*, *∆Fgatg22*, *∆Fgatg24* and *∆Fgatg26*, were significantly reduced (*P* < 0.05) (Fig. 4). Among them, the *∆Fgatg13*, *∆Fgatg22*, *∆Fgatg24* and *∆Fgatg26* mutants produced only (2.12 ± 0.56), (4.90 ± 1.30), (1.89 ± 0.33) and (4.38 ± 2.47) milligrams DON per kilogram inoculated wheat kernel powder, respectively, which was significantly less than that in PH-1 infections (Fig. 4). These results suggest that these genes are involved in positive regulation of DON biosynthesis in *F. graminearum*.

### Introduction of *FgATG1* and *FgATG5* into *M. oryzae ∆Moatg1* and *∆Moatg5* complements their phenotypic defects respectively

**Figure 5:**
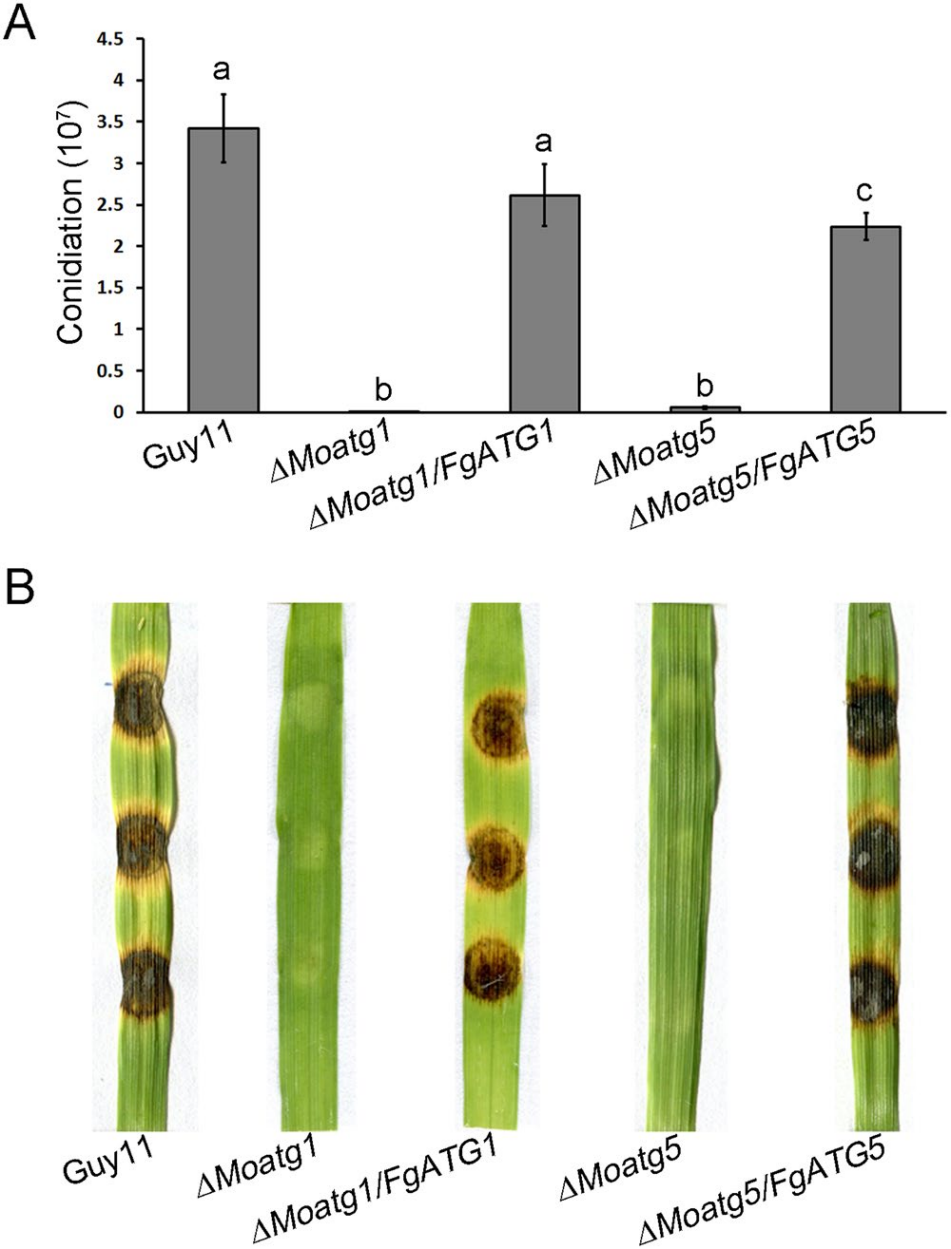
*FgATG1* and *FgATG5* complement the phenotypic defects of *M. oryzae ∆Moatg1* and *∆Moatg5* mutants respectively. (**A**) Bar chart showing the conidial production of the strains. Error bars in each column represent standard errors of three independent experiments. Different letters in each column indicate the significant difference of conidiation (*P* < 0.05). (**B**) Infection assays on the barley leaves. Leaves of 10-day-old barley seedlings were inoculated with mycelial plugs (0.5 cm) and examined at 7 days post inoculation (dpi). The complemented strains (*∆Moatg1/FgATG1* and *∆Moatg5/FgATG5*) were fully pathogenic to barley leaves.

It has been reported that *MoATG1* and *MoATG5* are necessary for conidiation, normal development and pathogenicity in the rice blast fungus *M. oryzae* ^18, 25^. To determine whether *FgATG1* and *FgATG5* can functionally complement defects in *∆Moatg1* and *∆Moatg5* mutants, the full length coding sequence of *FgATG1* (under the control of the *MoATG1* native promoter) and *FgATG5* (under the control of the *MoATG5* native promoter) were transformed into *∆Moatg1* and *∆Moatg5* mutants, respectively. The complementation transformants, *∆Moatg1*/*FgATG1* and *∆Moatg5*/*FgATG5*, were identified. Phenotypic analysis showed that *∆Moatg1* and *∆Moatg5* mutants cultured on CM plates for 12 days formed sparse aerial mycelium, but *∆Moatg1*/*FgATG1* and *∆Moatg5*/*FgATG5* strains produced dense aerial hyphae, which were identical to the wild-type strain Guy11 (data no shown). Compared to (3.42 ± 0.41) × 10^7^ conidia per plate produced by the wild-type strain Guy11, conidiogenesis in *∆Moatg1* and *∆Moatg5* mutants was significantly reduced (*P* < 0.05), only produced (7.73 ± 1.57) × 10^4^ and (5.35 ± 1.14) × 10^5^ conidia respectively. The *∆Moatg1*/*FgATG1* strain produced (2.62 ± 0.38) × 10^7^ conidia per plate approximately 76.6% of that of the wild-type strain (Fig. 5A). Under the same culture conditions, the *∆Moatg5*/*FgATG5* strain produced (2.24 ± 0.16) × 10^7^ conidia per plate, approximately 65.5% of that of the wild-type strains (Fig. 5A). The results suggest that introduction of *FgATG1* and *FgATG5* can restore the growth and conidiation of *∆Moatg1* and *∆Moatg5* mutants. To determine the pathogenicity of the *∆Moatg1*/*FgATG1* and *∆Moatg5*/*FgATG5* strains, the susceptible barley cv ZJ-8 was inoculated with mycelial plugs from each strain. Like the wild-type Guy11, the *∆Moatg1*/*FgATG1* and *∆Moatg5*/*FgATG5* strains caused severe disease on the barley leaves, while the *∆Moatg1* and *∆Moatg5* mutants failed to cause lesions (Fig. 5B). These results indicate that introduction of *FgATG1* and *FgATG5* can functionally complement the defects of *∆Moatg1* and *∆Moatg5* mutants, respectively.

### Autophagy is blocked in *∆Fgatg1* and *∆Fgatg5* mutants

**Figure 6:**
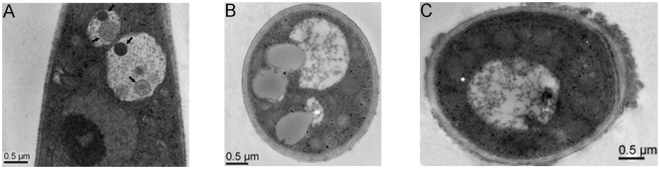
Observation of autophagic bodies in the hyphal vacuoles of the *∆Fgatg1* and *∆Fgatg5* mutants. (**A**) The vacuoles of PH-1 hyphal cells were filled with autophagic bodies. Arrows indicate the autophagic bodies. (**B**) and (**C**) Autophagic bodies were not observed in the vacuoles of the *∆Fgatg1* and *∆Fgatg5* mutants. Strains were cultured in liquid CM medium at 25 °C for 24 h in a 180 rpm shaker, and then shifted to liquid MM-N medium with 2 mM PMSF for 4 h. Vacuoles in the hyphae of these strains were observed using transmission electron microscopy. Scale bars = 0.5 µm.

To determine whether autophagy was affected by deletion of *ATG* genes in *F. graminearum*, the *∆Fgatg1* and *∆Fgatg5* mutants were used to observe autophagic bodies in vacuoles of hyphal cells by transmission electron microscopy. When strains were cultured in MM-N liquid medium in the presence of 2 mM PMSF for 4 h, autophagic bodies were observed clearly in the vacuoles of the wild-type strain PH-1 (Fig. 6A), while no autophagic bodies, or a few autophagosome-like structures were seen in vacuoles of *∆Fgatg1* and *∆Fgatg5* mutants (Fig. 6B,C). The results are consistent with the autophagic pathway being blocked in *∆Fgatg1* and *∆Fgatg5* mutants.

**Figure 7:**
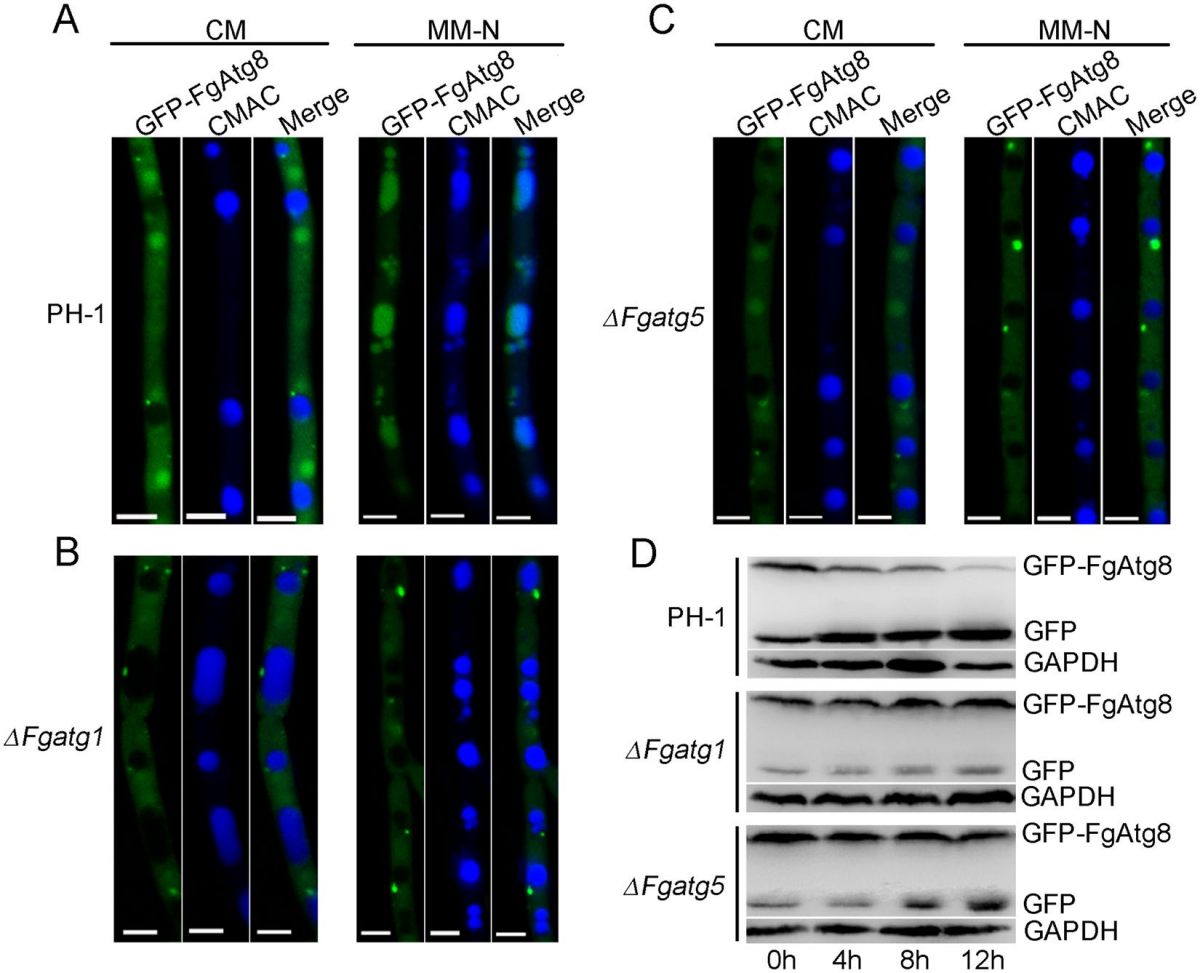
*FgATG1* and *FgATG5* were involved in autophagy in *F. graminearum*. (**A–C**) GFP-FgAtg8 localization in the PH-1, *∆Fgatg1* and *∆Fgatg5* mutants. PH-1 (**A**), *∆Fgatg1* (**B**) and *∆Fgatg5* (**C**) expressing GFP-FgAtg8 were grown in liquid CM medium at 25 °C for 24 h, and then shifted to liquid MM-N medium with 2 mM PMSF for 4 h. The vacuoles of hyphal cells of different strains were stained by CMAC (7-amino-4-chloromethylcoumarin) and examined by fluorescence microscopy. Scale bars = 5 µm. (**D**) GFP-FgAtg8 proteolysis assays of PH-1, *∆Fgatg1* and *∆Fgatg5*. Mycelia were harvested from liquid CM cultures after incubation in a 180 rpm-shaker in at 25 °C for 24 h. Autophagy was induced after nitrogen starvation for 4 h in MM-N liquid medium with 2 mM PMSF. Mycelia were collected at the indicated times and total proteins were extracted for the analysis of Western blotting by anti-GFP. Anti-GAPDH was shown as a control. Full-length blots were presented in Supplementary Figure S2.

The GFP-Atg8 processing assay can be used to monitor autophagosome delivery^44^. The GFP-FgAtg8 fusion protein was constructed and expressed in the PH-1 strain, *∆Fgatg1* and *∆Fgatg5* mutants, respectively. When the strains were cultured in rich-nutrient conditions (CM liquid medium) for 24 h, we observed that GFP-FgAtg8 was expressed and dispersed throughout the cytoplasm and GFP-FgAtg8 signals were localized in the punctate structures close to vacuoles in both PH-1 and *∆Fgatg1* and *∆Fgatg5* mutants (Fig. 7A,B and C). However, when strains were transferred to nitrogen starvation conditions (MM-N liquid medium) with 2 mM PMSF and incubated for 4 h, we observed that GFP-FgAtg8 accumulated in vacuoles of the wild-type PH-1 (Fig. 7A), while in hyphal cells of the *∆Fgatg1* and *∆Fgatg5* mutants, GFP-FgAtg8 remained outside vacuoles in punctate structures (Fig. 7B,C). These results indicate that autophagosomes in hyphal cells of *∆Fgatg1* and *∆Fgatg5* mutants are defective in fusion with vacuoles and that the autophagic pathway is blocked in *ATG* mutants.

To further investigate impairment of the *ATG* mutants in autophagic processing, we tested processing of GFP-FgAtg8 using proteolysis assays. Under non-induction conditions (0 h), a clear full-length GFP-FgAtg8 band (40 kDa) and a free GFP band (26 kDa) were detected in both PH-1 and *∆Fgatg1* and *∆Fgatg5* mutants by immunoblotting with an anti-GFP antibody (Fig. 7D). We observed that weak free-GFP bands were present in the *∆Fgatg1* and *∆Fgatg5* mutants (Fig. 7D), which suggested that the autophagy process was not completely blocked in the ∆*Fgatg1* and *∆Fgatg5* mutants. When hyphae of the wild-type PH-1 were shifted to nitrogen starvation conditions, an increasingly weaker GFP-FgAtg8 band, and an increasingly stronger free GFP band were detected as the induction time proceeded. By contrast, levels of full-length GFP-FgAtg8 in *∆Fgatg1* and *∆Fgatg5* mutants did not increase when the time of nitrogen-starvation was extended (Fig. 7D). This assay confirmed that GFP-FgAtg8 proteolysis was impaired in *∆Fgatg1* and *∆Fgatg5* mutants. Taken together, we conclude that the autophagy pathway is impaired in the *∆Fgatg1* and *∆Fgatg5* mutants and that this process is critical for fungal pathogenesis.

## Discussion

In this study, we identified 28 putative autophagy-related genes in *F. graminearum* using the known genome-wide set of *S. cerevisiae* Atg protein-encoding genes as a guide. Using targeted gene deletions, the predicted *F. graminearum ATG* genes were functionally characterized at a global scale. We found that autophagy is required for vegetative growth, asexual/sexual sporulation, DON production and pathogenicity in *F. graminearum*. In addition, we investigated the biological function of *FgATG1* and *FgATG5* and found that the autophagic process was blocked in *∆Fgatg1* and *∆Fgatg5* mutants. Taken together, we conclude that autophagy plays a critical role in many important physiological functions of *F. graminearum*.

Autophagy is a conserved process from budding yeasts to mammalian cells, and a major cellular pathway for degradation of long-lived proteins and cytoplasmic organelles^45–47^, important for maintaining homeostasis of cells. Autophagy is triggered by starvation stress, and leads to rearrangement of subcellular membranes to sequester cargo for delivery to the lysosome or vacuole, where sequestered material is then degraded and recycled^45, 48^. In addition to its homeostatic functions, autophagy is necessary for many aspects of development in multicellular organisms^49^. Previously, it has been observed that *ATG* mutants are impaired in mycelial growth and pathogenicity in several fungal species^17, 18, 50, 51^. In *F. graminearum*, deletion of *FgATG8* results in impairment of mycelial growth, usage of storage lipid droplets, formation of asexual/sexual spores and infection^20^. Similarly, *FgATG15* is involved in aerial hyphal growth, conidiogenesis, lipid droplet degradation and virulence^31^. In this study, the mutants of the 28 autophagy-related genes in *F. graminearum* were classified into two groups according to the rate of vegetative growth on PDA medium. Deletion of any of the genes in Group 1 resulted in a statistically significant difference in linear growth on the PDA plates compared to the wild-type strain PH-1 (Fig. 1A,B). However, the mutants of the *ATGs* in Group 2 had similar growth rates compared to an isogenic wild-type strain (Fig. 1C,D). This suggests that genes associated with Group 1 play wider roles in cellular viability and hyphal growth than those in Group 2. However, since multiple gene deletion mutants of Group 2 have not been generated and analyzed in the study, it is unknown whether genes of group 2 are redundant or functionally overlap. Therefore, we reasoned that further clarification of gene function in Group 2 proteins, for example in cellular viability and hyphal growth in future, would be necessary. Genes required presumptively for the selective pathways in *F. graminearum* are also found in both groups, such as *FgATG11*, *FgATG20*, *FgATG23*, *FgATG24* and *FgATG33* in Group 1 and *FgATG26*, *FgATG27*, *FgATG28* and *FgATG37* in Group 2, indicating that the two groups with different growing patterns had no close relation to selective and non-selective types of autophagy. Conidial production of 11 tested *ATG* mutants was analyzed statistically and showed that these mutants produced significantly less or even no conidia (Fig. 2A). Also, we found that perithecium development of *∆Fgatg1* and *∆Fgatg5* was completely blocked (Fig. 2B). Sporulation is associated closely with the energy metabolism of organisms^52^. Since autophagy provides nutrients by recycling cytoplasmic materials, a deficiency in this process probably reduces the production of conidia in consequence of the lack of energy and nutrients. Based on our pathogenicity assays, we found that most *ATG* mutants, but not *FgATG17*, exhibited a decrease in infection of wheat spikelets (Fig. 3). This affected not only symptom expression but also the ability of the fungus to spread to new spikelets. This suggests that the ability to colonise plant tissue is impaired, as well as the ability to produce spores to infect new hosts. Given these defects, it seems likely that autophagy is absolutely necessary for success of *F. graminearum* in the field and that the fungus would be unable to survive and cause disease without the operation of autophagy. Consistent with this loss of ability to colonise wheat tissue, we found that DON production of 13 tested *ATG* mutants was significantly reduced in comparison with PH-1 (Fig. 4), indicating that autophagy is necessary for fueling secondary metabolism in *F. graminearum*. However, the analysis of dynamic DON production for each of the ATG mutants was not carried out in the study. We cannot preclude at this stage that the lower levels of DON are associated with a delay in biosynthesis, rather than a reduction in the ability to synthesize the secondary metabolite. To date, at least 15 *TRI* genes in *F. graminearum* encoding trichothecene biosynthetic enzymes and regulators have been identified^53^. We found that the expression levels of *TRI5* (trichodiene synthase), *TRI6* (transcription regulator) and *TRI10* (transcription regulator) genes in mycelium cultured for 24 h were significantly decreased in *∆Fgatg20* and *∆Fgatg24* mutants (data not shown). The data suggest that reduction of DON biosynthesis of *ATG* mutants is associated with the low expression of the *TRI* genes in these mutants. Since deoxynivalenol (DON) was an important virulence determinant in *F. graminearum*, and DON is necessary to suppress plant defense enabling the pathogen to break through the rachis node^54^, the reduction of DON production in these mutants may be a cause of their loss of virulence or may be associated with the relative lack of infection ability of the mutants.

In *M. oryzae* autophagy genes can be classified into those predicted to be required for nonselective autophagy and those necessary for pexophagy, mitophagy, or the Cvt pathway^17^. Loss of any of the 16 genes necessary for nonselective macroautophagy leads to *M. oryzae* being unable to cause rice blast disease, but the 6 genes necessary only for selective autophagy are dispensable for appressorium-mediated plant infection^17^. However, in the present study, deletion of any of the genes required presumptively for pexophagy, mitophagy, or the Cvt pathway (*FgATG11*, *FgATG20*, *FgATG23*, *FgATG24*, *FgATG26*, *FgATG27*, *FgATG28*, *FgATG33* and *FgATG37*) and genes necessary for the nonselective macroautophagy (*FgATG1*, *FgATG2*, *FgATG3*, *FgATG4*, *FgATG5*, *FgATG6*, *FgATG7*, *FgATG8*, *FgATG9*, *FgATG10*, *FgATG12*, *FgATG13*, *FgATG14*, *FgATG15*, *FgATG16*, *FgATG18*, *FgATG22* and *FgATG29*) in *F. graminearum* leads to the significant reduction in virulence (Fig. 2). This suggests that both processes may be necessary for infection by *F. graminearum*. The functional difference of the *ATGs* in the two groups in plant infection between *M. oryzae* and *F. graminearum* may be due to DON production of these mutants in *F. graminearum*, which is significantly reduced and which plays such a critical role in plant infection. Interestingly, *∆Fgatg17* mutants were fully pathogenic, suggesting that this has a non-essential function in autophagy in *F. graminearum*. In *S. cerevisiae*, *∆atg17* mutants were almost completely defective in autophagy and produced few small autophagosomes that were less than half the normal size upon starvation^55, 56^. However, we found that *FgATG17* is not essential for GFP-FgAtg8 proteolysis and DON production in *F. graminearum* (Fig. S4).

In *S. cerevisiae*, Atg1 as a serine/threonine kinase involved in regulation of autophagy by protein phosphorylation^57^. Atg1 forms complexes with Atg13 and Atg17, which is required for the induction of autophagy^32^. Atg5 with Atg12 and Atg16 forms the Atg12-Atg5-Atg16 conjugation complex system and plays an essential role in the formation of autophagosomes^58–60^. In filamentous fungi, such as *Podospora anserina*, *Aspergillus fumigatus* and *M. oryzae*, homologs of *ATG1* and *ATG5* genes have been identified and characterized^18, 25, 61, 62^. We found that re-introduction of *FgATG1* and *FgATG5* into *M. oryzae Moatg1* and *Moatg5* mutants functionally complemented the phenotypes of the mutants, respectively, suggesting that Atg1 and Atg5 proteins in filamentous fungi probably play a conserved role in regulation of conidiogenesis and pathogenicity. We found that autophagic bodies in the *∆Fgatg1* and *∆Fgatg5* mutants were absent or obviously decreased under starvation conditions (Fig. 6). Moreover, disruption of *FgATG1* and *FgATG5* prevented movement of GFP-FgAtg8 to the vacuolar lumen when autophagy was induced (Fig. 7B,C). Consistent with this, impairment of autophagy in the *∆Fgatg1* and *∆Fgatg5* mutants was confirmed by GFP-FgAtg8 proteolysis assays ﻿(Fig. 7D). These results suggest that the autophagic pathway was mostly blocked in *∆Fgatg1* and *∆Fgatg5* mutants. However, the detailed mechanism of how *FgATG1* and *FgATG5* are involved in autophagy in *F*. *graminearum* requires further study. In *M. oryzae*, deletion of *MgATG1* gene influences the number of lipid bodies, and lipid storage in conidia in a *∆Mgatg5* mutant is reduced^18, 25^. In *Aspergillus oryzae*, AoAtg1 is involved in the Cvt pathway^28^. Hence, further studies will be necessary to reveal the relationship between autophagy and the lipid metabolism in *F. graminearum*.

In summary, we conclude that autophagy-related genes (except *FgATG17*) are involved in regulating vegetative growth, aerial mycelium formation, asexual/sexual sporulation, DON production and virulence in *F*. *graminearum*.

## Materials and Methods

### Fungal strains and culture conditions

The wild-type *F. graminearum* strain PH-1 and all derivative mutants in this study were cultured on PDA (potato dextrose agar, 200 g potato, 20 g dextrose, 20 g agar per 1 L water) plates at 25 °C to assess mycelial growth and colony characteristics. Conidiation assays of all strains were performed after growing 4 days in 1% mung bean liquid (MBL) medium (10 g mung beans boiled in 1 L water for 20 min). Cultures in PDB (PDA without agar) were used for genomic DNA isolation. Complete medium (CM) and minimal medium without the nitrogen source (MM-N) were used for autophagy assays.

### Generation of *ATG* deletion mutants

The DNA cassettes used for the gene deletions were constructed as described previously^63^. All PCR primers used in this study were listed in Table S2. The putative gene deletion mutants were identified and confirmed by PCR amplification and Southern blotting assays. Southern blot analysis was performed by the digoxigenin (DIG) high prime DNA labeling and detection starter Kit I (Roche, Mannheim, Germany).

### Phenotypic analysis

For the vegetative growth of each colony, 5-mm plugs cut from the edge of a 3-day-old colony of each strain were placed on PDA plates and incubated at 25 °C. After 3 days, the diameter of each strain was measured and recorded. For the conidiation assay, five 5-mm plugs of each strain from the edge of 3-day-old colony were inoculated in 20 mL 1% MBL. After 4 d cultivation in a 180 rpm-shaker, conidia of PH-1 were harvested by filtering through cheesecloth and directly counted using a haemocytometer. The conidia of mutants were harvested using the same method and then centrifuged at 8000 rpm for 10 min. The harvested conidia of mutants were re-suspended in 1 mL sterile distilled water and subsequently counted with the haemocytometer. Each experiment with three replicates was independently repeated three times. For self-crossing assays, perithecium formation was assayed on carrot agar (CA) medium as previously described^64^.

### Pathogenicity assays

Since the *F. graminearum ATG* mutants produced few conidia, mycelial plugs taken from the PDA plates were applied for pathogenicity assays. Agar plugs with mycelium from 3-day-old PDA plates of PH-1 or mutants were scraped off with dentiscalprum, and the middle spikelet of flowering wheat heads of the susceptible cultivar Jimai33 was inoculated. Symptomatic spikelets and quantification of infected spikelets among the whole wheat heads were determined after incubation for 14 days.

### DON production assays

After immersion in water for 12 h, 50 g wet wheat kernels were sterilized for 3 times and inoculated with ten mycelial plugs (5 mm in diameter) of each strain from the edge of 3-day-old colony and incubation at 25 °C for 25 days. The inoculated wheat kernels were dried at 37 °C for 24 h, and then broken into powder with juicer. The powder was delivered to the company named Pribolab biological engineering co., LTD in Qingdao which performed the DON production assays.

### Western blot analysis

A piece of agar blocks with mycelia of each tested strain was introduced into 20 mL of liquid CM medium. The suspension was shaken at 25 °C, 180 rpm for 24 h, and then transferred into the MM-N medium in the presence of 2 mM PMSF (phenymethylsulfonyl fluride) for 4 h, 8 h and 12 h. Hyphae of each sample were harvested, washed with sterile distilled water, ground into powder in liquid nitrogen, and then suspended in the protein lysis buffer (50 mM Tris-Cl (pH = 7.4), 0.15 M NaCl, 1 mM EDTA, 1% Triton × 100) added 1% 100 × Protease Inhibitor Cocktail for Fungal/Yeast Cell (Sangon, Shanghai, China). The lysate was centrifuged at 13200 rpm for 20 min at 4 °C after lysis for 30 min. Afterwards, 50 μL supernatant was mixed with isovolumetric 2 × protein loading buffer and boiled for 5 min and then cooled on the ice immediately. 10–15 μL of each sample was taken for loading on 10% SDS-PAGE gels. GFP-Tag (7G9) Mouse mAb (Abmart, Shanghai, China) as primary antibody was used at a 1:5000 dilution. HiSec^TM^ HRP-conjugated Goat Anti-Mouse IgG (H + L) (Vazyme, Nanjing, China) was applied to immunoblot analysis at a 1:10000–1:50000 dilution. FD^TM^ FDbio-Femto Ecl chemiluminescent substrate (Fdbio science, Hangzhou, China) was used for antigen antibody detections.

### Complementation analysis of *Moatg1* and *Moatg5* by introducing *FgATG1* and *FgATG5* respectively

The pCB1532-FgATG1 and pCB1532-FgATG5 vectors for the complementation of *Moatg1* and *Moatg5* mutants respectively were constructed as described previously^63^.

### Confocal microscopy and transmission electron microscopy assays

Hyphae expressing the GFP-Atg8 fusion protein were cultured in CM medium at 25 °C, 180 rpm for 24 h, and then transferred into the MM-N medium in the presence of 2 mM PMSF for 4 h at 25 °C in a 180 rpm shaker. CMAC (7-amino-4-chloromethycoumarin) (Invitrogen, USA) was used for vacuole staining. Photographs were taken under a confocal laser scanning microscopy. Transmission electron microscopy was implemented as described previously^18^.

### Data Availability

All data generated or analysed during this study are including in this published article (and its Supplementary Information files).

## Electronic supplementary material

Supplementary Information
