# Supplementary material for: Genome-wide functional analysis reveals that autophagy is necessary for growth, sporulation, deoxynivalenol production and virulence in Fusarium graminearum
Source: Sci Rep. 2017 Sep 11;7:11062. doi: 10.1038/s41598-017-11640-z (PMC5594004; doi:10.1038/s41598-017-11640-z)
Supplement: Supplementary file 1 — Supplementary Information [file 41598_2017_11640_MOESM1_ESM.pdf]

Genome-wide functional analysis reveals that autophagy is necessary for growth, sporulation, deoxynivalenol production and virulence in *Fusarium graminearum*

Wuyun Lv[1], Chunyan Wang[1], Nan Yang[1], Yawei Que[1], Nicholas J. Talbot[2] and Zhengyi Wang[1*]

[1]State Key Laboratory for Rice Biology, Institute of Biotechnology, Zhejiang University, Hangzhou 310029, China

[2] School of Biosciences, University of Exeter, Geoffrey Pope Building, Exeter EX4 4QD, United Kingdom

*Corresponding author: Zhengyi Wang; E-mail: zhywang@zju.edu.cn

Supporting information

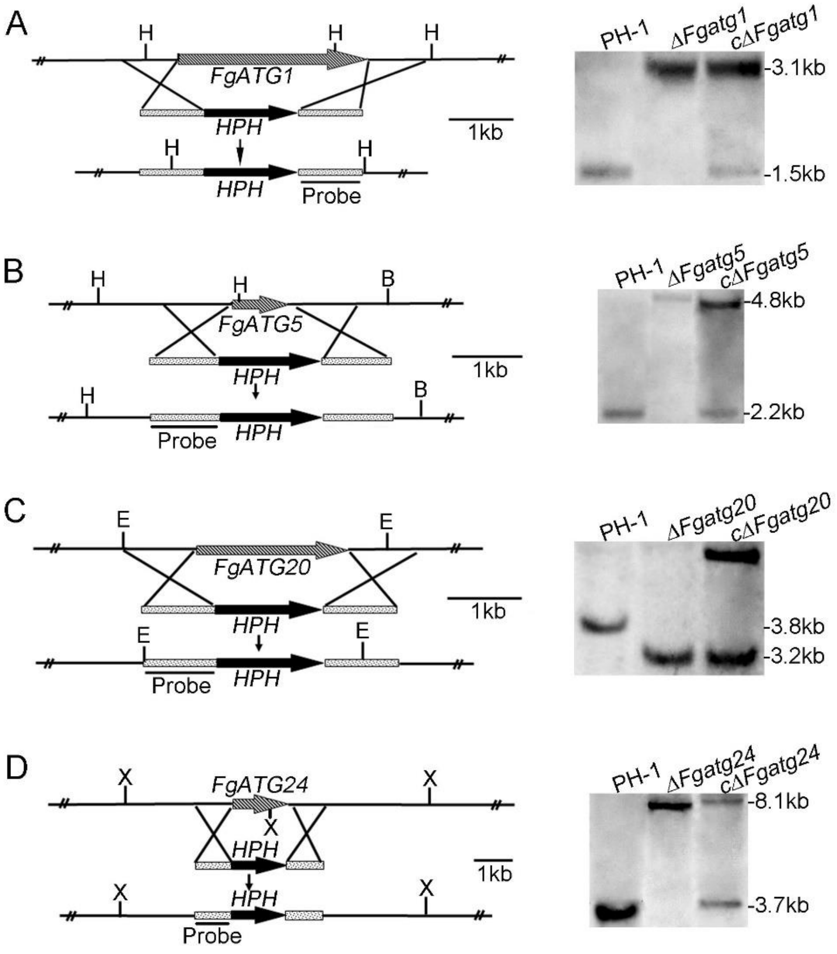

Figure S1

Figure S1. Targeted gene replacements of *FgATG1*, *FgATG5*, *FgATG20* and *FgATG24*. (A) Construction of the DNA fragment for *FgATG1* knockout and Southern blot analysis. Genomic DNAs of the wild-type PH-1, Δ*Fgatg1* mutant and the complemented strain cΔ*Fgatg1* were digested with *Hin*d Ⅲ and probed with a ~1.0kb fragment amplified with the primers 1-PF and 1-PR. H=*Hin*d Ⅲ. (B) Construction of the DNA fragment for *FgATG5* knockout and Southern blot analysis. Genomic DNAs of PH-1, Δ*Fgatg5* mutant and the complemented strain cΔ*Fgatg5* were digested with *Hin*d Ⅲ and *Bam*H Ⅰ and probed with a ~1.0kb fragment amplified with the

primers 5-PF and 5-PR. H=*Hin*d Ⅲ; B=*Bam*HⅠ. (C) Construction of the DNA fragment for *FgATG20* knockout and Southern blot analysis. Genomic DNAs of PH-1, Δ*Fgatg20* mutant and the complemented strain cΔ*Fgatg20* were digested with *Eco*RⅠ and probed with a ~1.0kb fragment amplified with the primers 20-PF and 20-PR. E=*Eco*RⅠ. (D) Construction of the DNA fragment for *FgATG24* knockout and Southern blot analysis. Genomic DNAs of PH-1, Δ*Fgatg24* mutant and the complemented strain cΔ*Fgatg24* were digested with *Xba*Ⅰ and probed with a ~1.0kb fragment amplified with the primers 24-PF and 24-PR. X=*Xba*Ⅰ. The complementation of the Δ*Fgatg1*, Δ*Fgatg5*, Δ*Fgatg20* and Δ*Fgatg24* mutants was performed by re-introduction of *FgATG1*, *FgATG5*, *FgATG20* and *FgATG24* genes under their own promoters and terminators, respectively.

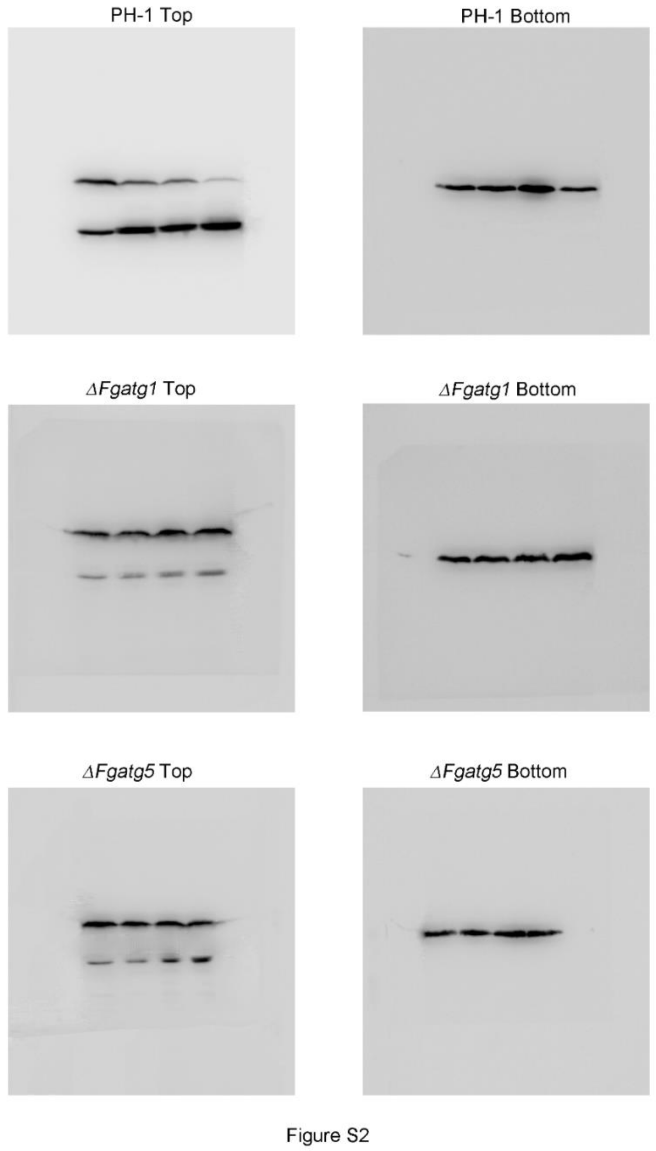

Figure S2. The original full-length blots of Figure 7D. GFP-FgAtg8 proteolysis assays of PH-1, Δ*Fgatg1* and Δ*Fgatg5*. Mycelia were harvested from liquid CM cultures after incubation in a 180 rpm-shaker in at 25℃ for 24 h. Autophagy was induced after nitrogen starvation for 4 h in MM-N liquid medium with 2 mM PMSF. Mycelia were collected at 0, 4, 8 and 12 h respectively and total proteins were extracted for the analysis of Western blotting by anti-GFP. Anti-GAPDH was shown as a control (see Figure 7D).

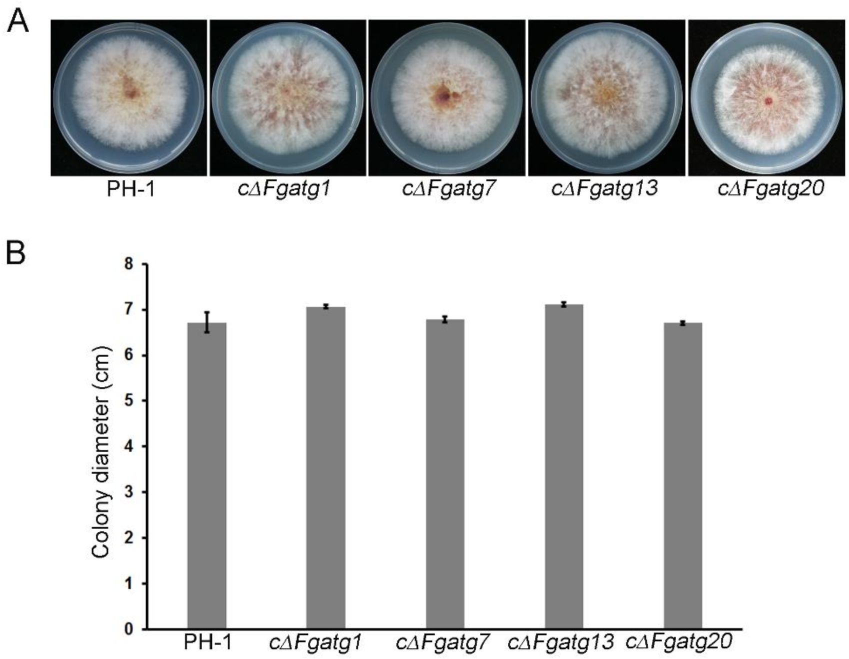

Figure S3

Figure S3. The defects in vegetative growth of the deletion mutants could be complemented by re-introduction of the corresponding *ATG* genes. (A) Colonies of wild-type PH-1 and the complemented strains *c*Δ*Fgatg1*, *c*Δ*Fgatg7*, *c*Δ*Fgatg13* and *c*Δ*Fgatg20*. Photographs were taken after incubation on PDA plates at 25℃ for 3 days. (B) Bar chart showing colony diameters of PH-1 and the complemented strains *c*Δ*Fgatg1*, *c*Δ*Fgatg7*, *c*Δ*Fgatg13* and *c*Δ*Fgatg20*. Linear bars in each column denote standard errors of three experiments.

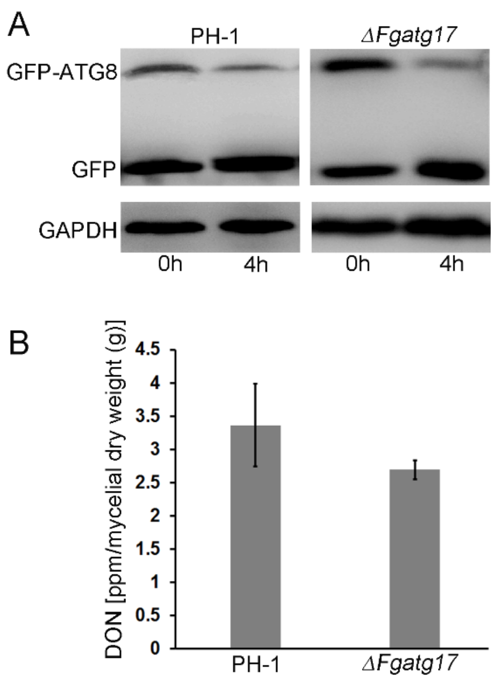

Figure S4

Figure S4. The *FgATG17* role in the autophagy and DON production of *F. graminearum*. (A) GFP-FgAtg8 proteolysis assays of PH-1 and Δ*Fgatg17*. Mycelia were harvested from liquid CM cultures after incubation for 24 h and from MM-N liquid medium with 2 mM PMSF for 4 h in a 180 rpm-shaker in at 25℃. Total proteins were extracted for the analysis of Western blotting by anti-GFP. Anti-GAPDH was shown as a control. (B) DON production in PH-1 and Δ*Fgatg17*. There was no significant difference in level of DON production between PH-1 and Δ*Fgatg17* mutant. Methods Enzyme-linked immunosorbent assay (ELISA) was used to determine the level of DON production of in PH-1 and Δ*Fgatg17*. Filter liquor containing DON was got from the 25 mL TBI liquid medium with five 5-mm mycelial plugs after incubation at 28℃ for 4 days in a 150 rpm shaker. Linear bars in each column denote standard errors of three repeats.

Table S1. Autophagy-related genes in *Fusarium graminearum*

**Yeast**			***Fusarium graminearum***
**Gene name**	**Systematic name**	**Function**	**Gene**
ATG1	YGL180W	Protein serine/threonine kinase; required for vesicle formation in autophagy and the cytoplasm-to-vacuole targeting (Cvt) pathway	FGSG_05547
ATG2	YNL242W	Peripheral membrane protein required for autophagic vesicle formation; involved in Atg9p cycling between the phagophore assembly site and mitochondria	FGSG_10283
ATG3	YNR007C	E2-like enzyme; involved in autophagy and the Cvt pathway; plays a role in formation of Atg8p-phosphatidylethanolamine conjugates	FGSG_08900
ATG4	YNL223W	Conserved cysteine protease required for autophagy; cleaves Atg8p to a form required for autophagosome and Cvt vesicle generation	FGSG_09858
ATG5	YPL149W	Undergoes conjugation with Atg12p to form a complex involved in Atg8p lipidation	FGSG_10053
ATG6	YPL120W	Subunit of phosphatidylinositol (PtdIns) 3-kinase complexes I and II	FGSG_11805
ATG7	YHR171W	Mediates the attachment of Atg12p to Atg5p and Atg8p to phosphatidylethanolamine	FGSG_10226
ATG8	YBL078C	Component of autophagosomes and Cvt vesicles; unique ubiquitin-like protein conjugated to PE involved in phagophore expansion	FGSG_10740
ATG9	YDL149W	Transmembrane protein involved in forming Cvt and autophagic vesicles; cycles between the phagophore assembly site (PAS) and other cytosolic punctate structures	FGSG_13660
ATG10	YLL042C	Conserved E2-like conjugating enzyme; mediates formation of the Atg12p-Atg5p conjugate	FGSG_00611
ATG11	YPR049C	Required for delivery of aminopeptidase I to the vacuole in Cvt pathway; involved in pexophagy and mitophagy	FGSG_00382
ATG12	YBR217W	Ubiquitin-like modifier involved in autophagy and the Cvt pathway; conjugated to Atg5p to form a complex involved in Atg8p lipidation	FGSG_13550
ATG13	YPR185W	Regulatory subunit of the Atg1p signaling complex; stimulates Atg1p kinase activity	FGSG_08491
ATG14	YBR128C	Autophagy-specific subunit of phosphatidylinositol 3-kinase complex I	FGSG_00675[a]
ATG15	YCR068W	Lipase required for intravacuolar lysis of autophagic and Cvt bodies	FGSG_02519
ATG16	YMR159C	Interacts with Atg12p-Atg5p conjugates to form Atg12p-Atg5p-Atg16p multimers	FGSG_02566
ATG17	YLR423C	Scaffold protein responsible for phagophore assembly site organization; regulatory subunit of an autophagy-specific complex that includes Atg1p and Atg13p	FGSG_06510
ATG18	YFR021W	Phosphoinositide binding protein; required for vesicle formation in autophagy and the Cvt pathway	FGSG_04297
ATG19	YOL082W	Receptor protein for the Cvt pathway; delivers cargo proteins aminopeptidase I and alpha-mannosidase to the phagophore assembly site for packaging into Cvt vesicles	No homolog found

ATG20	YDL113C	Sorting nexin family member; required for the Cvt pathway and for endosomal sorting	FGSG_06950
ATG21	YPL100W	Phosphoinositide binding protein; required for vesicle formation in the Cvt pathway	No homolog found
ATG22	YCL038C	Vacuolar integral membrane protein required for efflux of amino acids during autophagic body breakdown in the vacuole	FGSG_01225
ATG23	YLR431C	Peripheral membrane protein required for autophagy and the Cvt pathway	FGSG_02793[a]
ATG24	YJL036W	Sorting nexin; involved in the Cvt pathway; forms complexes with Snx41p and with Atg20p	FGSG_09157
ATG26	YLR189C	UDP-glucose:sterol glucosyltransferase	FGSG_13231
ATG27	YJL178C	Type I membrane protein involved in autophagy and the Cvt pathway	FGSG_01574[a]
ATG28	XP_001821 228	Involved in pexophagy	FGSG_07526[a,b]
ATG29	YPL166W	Required for recruiting other ATG proteins to the pre-autophagosomal structure (PAS); interacts with Atg17p	FGSG_13575[a]
ATG30 [c]	CCA39286	Pexophagy receptor	No homolog found
ATG31	YDR022C	Forms a complex with Atg17p and Atg29p that localizes other proteins to the pre-autophagosomal structure	No homolog found
ATG32	YIL146C	Mitophagy protein	No homolog found
ATG33	YLR356W	Required for mitophagy	FGSG_00549[a]
ATG34	YOL083W	Receptor protein involved in the transport of Ams1p	No homolog found
ATG35 [c]	C4QVX6	A micropexophagy-specific protein	No homolog found
ATG36	YJL185C	Pexophagy receptor, interacts with Pex3p	No homolog found
ATG37	C4R8D7	Involved in pexophagy	FGSG_05200
ATG38	YLR211C	Homodimeric subunit of autophagy-specific PtdIns-3-kinase complex I	No homolog found
ATG39	YLR312C	Autophagy receptor with a role in degradation of the ER and nucleus	No homolog found
ATG40	YOR152C	Involved specifically in autophagy of cortical and cytoplasmic ER	No homolog found
ATG41	YPL250C	Involved in autophagosome formation	No homolog found

The systematic names of *S. cerevisiae* genes and their functions were obtained from the National Center for Biotechnology Information database (http://www.ncbi.nlm.nih.gov/). Most homologs of *F. graminearum* were identified by BLAST searching with ATG gens in *S. cerevisiae*, but some were identified by the comparison to the *ATGs* in *Magnaporthe oryzae* [a], *Aspergillus oryzae* [b] and *Pichia pastoris* [c], respectively. ATG30 and ATG35 have been previously described in *P. pastoris* but not in *S. cerevisiae*.

Table S2. PCR primers used in this study.

Primer	Sequence (5’-3’)	Relevant characteristics
1-UF	GCAGCCATGTACCAGCAT	Amplification of upstream fragment of *FgATG1* for the construction of deletion mutants
1-UR	TCCACTAGCTCCAGCCAAGGGCATGTAAACGGAGGAC	
1-DF	AGTAGATGCCGACCGAACAAGCCCAGGAGGCGGATTT	Amplification of downstream fragment of *FgATG1* for the construction of deletion mutants
1-DR	TCCCGCACGGTGTTAGAT	
1-NF	CTCTTCTGCCTGTAGTGC	Amplification of the final products using for the *FgATG1* gene deletion
1-NR	CACGAAGAACAAAGACGA	
1-YF	CTTTACAGCGAAATCCAA	For identification of *FgATG1* gene deletion transformants
1-YR	GGGAAGTAGAGGGAAGCA	
1-PF	GGGGTTTGGCTTGCGACTC	Amplification of *FgATG1* probe using for Southern blot analysis
1-PR	TCCCGCACGGTGTTAGAT	
2-UF	GTTGCCAATGAGTTGAGTC	Amplification of upstream fragment of *FgATG2* for the construction of deletion mutants
2-UR	TCCACTAGCTCCAGCCAAGAAGGGCATGTTTAGAAGAA	
2-DF	AGTAGATGCCGACCGAACACTGGATGGGACAGCAAGA	Amplification of downstream fragment of *FgATG2* for the construction of deletion mutants
2-DR	ACATCGCAACACTCAACG	
2-NF	CTTCTGTCCGTCGCTCAA	Amplification of the final products using for the *FgATG2* gene deletion
2-NR	TACAATGCGCTCAAGACG	
2-YF	AATCCAAGCCGAGTCGTT	For identification of *FgATG2* gene deletion transformants
2-YR	GTTTGCCCTCCTTGTGAA	
3-UF	CTCGGCGGACCGTAGCGTA	Amplification of upstream fragment of *FgATG3* for the construction of deletion mutants
3-UR	TCCACTAGCTCCAGCCAAGAAGGGTGCGGGGTTGTTA	
3-DF	AGTAGATGCCGACCGAACACACATGCCACCAGAAAACAC	Amplification of downstream fragment of *FgATG3* for the construction of deletion mutants
3-DR	CCAGACACCAAACGGAACA	
3-NF	CGACAAGGAATGGGAGGA	Amplification of the final products using for the *FgATG3* gene deletion
3-NR	TGCGTGCCAGAGTCAGCT	

3-YF	CAACCTTCCGCCAGACCG	For identification of *FgATG3* gene deletion transformants
3-YR	GCGAGATAGCCTGACAGATACA	
4-UF	TTGATTCGCTCCGTCCAT	Amplification of upstream fragment of *FgATG4* for the construction of deletion mutants
4-UR	TCCACTAGCTCCAGCCAAGGGTAACAGGCAAGGCAGAC	
4-DF	AGTAGATGCCGACCGAACAGGCTAAGGATAAGAAGCGACC	Amplification of downstream fragment of *FgATG4* for the construction of deletion mutants
4-DR	GTCTGACGAAAATGGAGTGG	
4-NF	TCAACGGGGTGGTAAACG	Amplification of the final products using for the *FgATG4* gene deletion
4-NR	GCCAGCAAATACAAGTCGG	
4-YF	CACAGCTCAACCGCCTAAT	For identification of *FgATG4* gene deletion transformants
4-YR	CGTGGGAGTTTGTCAATGCT	
5-UF	CGCAAGCCAAGGACCATC	Amplification of upstream fragment of *FgATG5* for the construction of deletion mutants
5-UR	TCCACTAGCTCCAGCCAAGAGTGAGGCAAGGCGTGATA	
5-DF	AGTAGATGCCGACCGAACACCTCCTCCTCGGCGATAT	Amplification of downstream fragment of *FgATG5* for the construction of deletion mutants
5-DR	TGGGTCTGCGATACAAGCT	
5-NF	CTACCCTTGAGCCTCTTACATC	Amplification of the final products using for the *FgATG5* gene deletion
5-NR	CGAGCATATCCTTGTGAGTGG	
5-YF	CACCGACGACTCCTTTCAT	For identification of *FgATG5* gene deletion transformants
5-YR	GGTTTGCGTTGCCATTTC	
5-PF	TTCGCCTTTCATGTTCCC	Amplification of *FgATG5* probe using for Southern blot analysis
5-PR	ATTCGCTGCCGTTAGAGC	
6-UF	CCTGTTGCCATGTCGATTTC	Amplification of upstream fragment of *FgATG6* for the construction of deletion mutants
6-UR	TCCACTAGCTCCAGCCAAGGCCTTAGCGGTGACGTTGT	
6-DF	AGTAGATGCCGACCGAACACATTAGGGGTAGTAGACTTGCG	Amplification of downstream fragment of *FgATG6* for the construction of deletion mutants
6-DR	AACGGTTCCTGGGTTGCT	
6-NF	GCATTCACCCGCTTTAGT	Amplification of the final products using for the *FgATG6* gene deletion
6-NR	CTGGGTTACTGGTGAGGT	

6-YF	AGTTATCCGCATCATGTCTCG	For identification of *FgATG6* gene deletion transformants
6-YR	CCTGTCCATTTCGCTTCC	
7-UF	GTTTGTTTGTGAGGACGGG	Amplification of upstream fragment of *FgATG7* for the construction of deletion mutants
7-UR	TCCACTAGCTCCAGCCAAGCTGCTCGGGGTTGAATGT	
7-DF	AGTAGATGCCGACCGAACACGTTGCTGGGTTTACTTTGC	Amplification of downstream fragment of *FgATG7* for the construction of deletion mutants
7-DR	TATCGTGCGTTGCGTTTC	
7-NF	ATGCTGCCGGAACTGTATC	Amplification of the final products using for the *FgATG7* gene deletion
7-NR	CAGCCTTTCGCAGTATCTTC	
7-YF	TTCACTGACCCTTCGACATACC	For identification of *FgATG7* gene deletion transformants
7-YR	CCCAACGCAGCATTCATAAC	
8-UF	AAGGAGCGTGTCTAGGGTGG	Amplification of upstream fragment of *FgATG8* for the construction of deletion mutants
8-UR	TCCACTAGCTCCAGCCAAGCCAGAGTTCATCGACGAGTTTC	
8-DF	AGTAGATGCCGACCGAACATCACGAACGCCCGAACAA	Amplification of downstream fragment of *FgATG8* for the construction of deletion mutants
8-DR	CCAAAATCCCCTCCAAAAGC	
8-NF	AGGTTTCCTGGTAGCGAGTG	Amplification of the final products using for the *FgATG8* gene deletion
8-NR	GGTCAACAAGGCTCATTCG	
8-YF	CTTACCGCCTCCACAACAA	For identification of *FgATG8* gene deletion transformants
8-YR	CTATGTGACAACGCCAACG	
9-UF	CGTTAGGGGCGTGAGACC	Amplification of upstream fragment of *FgATG9* for the construction of deletion mutants
9-UR	TCCACTAGCTCCAGCCAAGGTGCGGAAGAATGACAAGG	
9-DF	AGTAGATGCCGACCGAACATCCCCTAATACCATGCCC	Amplification of downstream fragment of *FgATG9* for the construction of deletion mutants
9-DR	CACGCACTTACTGTAAAGCACC	
9-NF	GGAGGTTGTGGGTGGTTATC	Amplification of the final products using for the *FgATG9* gene deletion
9-NR	CCAGGGCAAAGGCTGTTA	
9-YF	ACCCGAGGGAGAAAGCAC	For identification of *FgATG9* gene deletion transformants
9-YR	GCTGTCTCAACTTCTGGCTCA	

10-UF	CGAAGGAGGTATGGGGAGC	Amplification of upstream fragment of *FgATG10* for the construction of deletion mutants
10-UR	TCCACTAGCTCCAGCCAAGCGAGCAGTATTGAAGTCGTGGT	
10-DF	AGTAGATGCCGACCGAACAGCCTAACTAACACCTTGCTCAC	Amplification of downstream fragment of *FgATG10* for the construction of deletion mutants
10-DR	CCGCTTTGTACTTGCTCTTC	
10-NF	CGGAATAAGGATAGAAGGCACG	Amplification of the final products using for the *FgATG10* gene deletion
10-NR	TGCTGGATGTTGTCGCTCT	
10-YF	CCGACTTTTCTTATGGTGGAGG	For identification of *FgATG10* gene deletion transformants
10-YR	CATCAGCGGGTAGGTTGC	
11-UF	CGCCGCTACCAACTATTA	Amplification of upstream fragment of *FgATG11* for the construction of deletion mutants
11-UR	TCCACTAGCTCCAGCCAAGGATTGACCGCCACAGATA	
11-DF	AGTAGATGCCGACCGAACATGGGATTCTTTGTGGAGT	Amplification of downstream fragment of *FgATG11* for the construction of deletion mutants
11-DR	AAATCGCTTAGGCAACTC	
11-NF	TCGTCAACGCCTTCCATA	Amplification of the final products using for the *FgATG11* gene deletion
11-NR	TCTTCTCGGCTGGTCCCT	
11-YF	ACGAATTTACATACAGAGGT	For identification of *FgATG11* gene deletion transformants
11-YR	ATTTCCCGTCCAGTCATA	
12-UF	GCACCTGTTACGCAATAT	Amplification of upstream fragment of *FgATG12* for the construction of deletion mutants
12-UR	TCCACTAGCTCCAGCCAAGGCAGAAGCCGTCCTCTTA	
12-DF	AGTAGATGCCGACCGAACATTTGCGATGATGGAAAGT	Amplification of downstream fragment of *FgATG12* for the construction of deletion mutants
12-DR	TTGTCGGTGATAGTCTTGC	
12-NF	CGATGTCCAGTCTCGCAGTA	Amplification of the final products using for the *FgATG12* gene deletion
12-NR	GTTGCCCTTGGTATGGAA	
12-YF	CTGCCTCACCAATGCCTCT	For identification of *FgATG12* gene deletion transformants
12-YR	CATCTTGGGCCAACAACG	

13-UF	GAGGTCCTGAGTGGTGAC	Amplification of upstream fragment of *FgATG13* for the construction of deletion mutants
13-UR	TCCACTAGCTCCAGCCAAGAGATTTCGACGGTCTTATC	
13-DF	AGTAGATGCCGACCGAACAGCAGCGAACAGTGGACAG	Amplification of downstream fragment of *FgATG13* for the construction of deletion mutants
13-DR	GTTAGGCAGGCTGGGTTA	
13-NF	GGCTCAAACGAAACAATC	Amplification of the final products using for the *FgATG13* gene deletion
13-NR	AGCGATCAACTTAACTGC	
13-YF	TGATACAATCCCATTTACAG	For identification of *FgATG13* gene deletion transformants
13-YR	GCTCAACTTGCCACCTCT	
14-UF	GGCTGCCCAGACTTTGAA	Amplification of upstream fragment of *FgATG14* for the construction of deletion mutants
14-UR	TCCACTAGCTCCAGCCAAGCACTTGGTGCGAAGTAGTTGTC	
14-DF	AGTAGATGCCGACCGAACAGGGTCCTTCCTTGACAAATC	Amplification of downstream fragment of *FgATG14* for the construction of deletion mutants
14-DR	AATCCCAGCCAACCTCTTC	
14-NF	CAGTCAAACGGCTCAAGAAC	Amplification of the final products using for the *FgATG14* gene deletion
14-NR	CGCCCGTCACTCCTCAAC	
14-YF	CGCTGCTGTATCTGATGGTC	For identification of *FgATG14* gene deletion transformants
14-YR	GGGGATAGTCTCGATGAGGG	
15-UF	TGGAGAACTCAGCCTTGC	Amplification of upstream fragment of *FgATG15* for the construction of deletion mutants
15-UR	TCCACTAGCTCCAGCCAAGGTACCTGGCACCTACAAAT	
15-DF	AGTAGATGCCGACCGAACACAGGTAGCCCTGGAGCAT	Amplification of downstream fragment of *FgATG15* for the construction of deletion mutants
15-DR	CGATTCGGACTTTCTTGC	
15-NF	ATGTTATTGTGATCGAGGGT	Amplification of the final products using for the *FgATG15* gene deletion
15-NR	GACGCTCCTTTGCCTGCT	
15-YF	GTCACTATCACGCAGCAAT	For identification of *FgATG15* gene deletion transformants
15-YR	ATCAGCTAAACGGTGGATT	

16-UF<br>16-UR	AAAATAGTTGGCAGACATAC<br>TCCACTAGCTCCAGCCAAGCAACCTCCACTTGTATCG	Amplification of upstream fragment of *FgATG16* for the construction of deletion mutants
16-DF<br>16-DR	AGTAGATGCCGACCGAACAAGTCTAAGGAACAACGCTC<br>GCATCCAACAGAAACATC	Amplification of downstream fragment of *FgATG16* for the construction of deletion mutants
16-NF<br>16-NR	CGCCACAGTGCTAAGAGG<br>CTTCTACACCCATTCATCTC	Amplification of the final products using for the *FgATG16* gene deletion
16-YF<br>16-YR	CATTATGCGACATCATCC<br>GTTTCTCAGCGACACCTT	For identification of *FgATG16* gene deletion transformants
17-UF<br>17-UR	TTCAACGCCTACCATTCG<br>TCCACTAGCTCCAGCCAAGCGGCTCCACTTCCTTCTA	Amplification of upstream fragment of *FgATG17* for the construction of deletion mutants
17-DF<br>17-DR	AGTAGATGCCGACCGAACAACGATTGATAGCGAGGGT<br>CTGCTTGCTGGGTCTTTT	Amplification of downstream fragment of *FgATG17* for the construction of deletion mutants
17-NF<br>17-NR	GAGCGTATTAGACAAGATTC<br>CCTTCTCGCCTACCTCTA	Amplification of the final products using for the *FgATG17* gene deletion
17-YF<br>17-YR	CGAAGCCTTCTTGACTTTAT<br>CGTTGCCTTGGGTCTGTG	For identification of *FgATG17* gene deletion transformants
18-UF<br>18-UR	GCCTCATTTATTTCGTCT<br>TCCACTAGCTCCAGCCAAGCTGATTTGAAGCGTATGTTA	Amplification of upstream fragment of *FgATG18* for the construction of deletion mutants
18-DF<br>18-DR	AGTAGATGCCGACCGAACACGTTTATCGGATTGGGTTTA<br>TCATCTTCGCTTTGCCTA	Amplification of downstream fragment of *FgATG18* for the construction of deletion mutants
18-NF<br>18-NR	TAGCAGGCAGGGACTCAG<br>TACTCCCGTTGAAGCGAC	Amplification of the final products using for the *FgATG18* gene deletion
18-YF<br>18-YR	GGGACTAATTGCGGTTCT<br>ACAGTTCTCGGAGGATGG	For identification of *FgATG18* gene deletion transformants

20-UF<br>20-UR	TTGCACGTTAAGATAAGC<br>TCCACTAGCTCCAGCCAAGGGAGTGATACGAGGATGAA	Amplification of upstream fragment of *FgATG20* for the construction of deletion mutants
20-DF<br>20-DR	AGTAGATGCCGACCGAACATTTGGAGCTTGAAGCAGT<br>GCGATGGAGGTGATAGGC	Amplification of downstream fragment of *FgATG20* for the construction of deletion mutants
20-NF<br>20-NR	TGCGTTATCTATCCCTTGA<br>GTGCCTTTGTAAGTAGCG	Amplification of the final products using for the *FgATG20* gene deletion
20-YF<br>20-YR	CCGGCCTTGAATGCTAAC<br>TAGGGTGAAGACGAGTAAGA	For identification of *FgATG20* gene deletion transformants
20-PF<br>20-PR	TATGCTTGGTCAGGGTATGG<br>TAAGCTGGAACCTGGGAAG	Amplification of *FgATG20* probe using for Southern blot analysis
22-UF<br>22-UR	CTCCACTTCCCCTCATTTTC<br>TCCACTAGCTCCAGCCAAGGGCAACGCTCAACACCAG	Amplification of upstream fragment of *FgATG22* for the construction of deletion mutants
22-DF<br>22-DR	AGTAGATGCCGACCGAACAGTGGGAGTCCAGAATGCG<br>GGTCTTGAAGGTGCCTGTTG	Amplification of downstream fragment of *FgATG22* for the construction of deletion mutants
22-NF<br>22-NR	GTTTCCTGAATACCCAAGC<br>GTGGCGTTCCTGTTCTGC	Amplification of the final products using for the *FgATG22* gene deletion
22-YF<br>22-YR	CAAATATCTCAAAACGGTC<br>CAATACATTGTCCATCGC	For identification of *FgATG22* gene deletion transformants
23-UF<br>23-UR	CGCTAAATCAGGGGAGAATG<br>TCCACTAGCTCCAGCCAAGTCTTAGGGGACCAAGGGAC	Amplification of upstream fragment of *FgATG23* for the construction of deletion mutants
23-DF<br>23-DR	AGTAGATGCCGACCGAACATTTCCTCCGCTGCGTTTC<br>GAGATACCGGCGTTGTTGAT	Amplification of downstream fragment of *FgATG23* for the construction of deletion mutants
23-NF<br>23-NR	CGATGCTCACTCGGCTCT<br>CGGCGTTGTTGATGAGGAT	Amplification of the final products using for the *FgATG23* gene deletion

23-YF<br>23-YR	TGCTACCCCATTGCCTCC<br>CGTTAGACTCCTTCGTTTCG	For identification of *FgATG23* gene deletion transformants
24-UF<br>24-UR	TTATTGAAGATGAGGCAGAG<br>TCCACTAGCTCCAGCCAAGAATGCCGCAGTTGTAGGT	Amplification of upstream fragment of *FgATG24* for the construction of deletion mutants
24-DF<br>24-DR	AGTAGATGCCGACCGAACAGAGGGATGATGGATGACTG<br>GATGACGACCATGACAAAA	Amplification of downstream fragment of *FgATG24* for the construction of deletion mutants
24-NF<br>24-NR	GTCCAAGGAGCAGAAGTAG<br>TCAAGCACCCAGACCTCC	Amplification of the final products using for the *FgATG24* gene deletion
24-YF<br>24-YR	CGAAGAACCAGGCCATGA<br>GGACAAGCGGCTGAGGAA	For identification of *FgATG24* gene deletion transformants
24-PF<br>24-PR	ACGGTCAAGGCTCAGGTG<br>TTGTAGGTTGGAGGAAGTGC	Amplification of *FgATG24* probe using for Southern blot analysis
26-UF<br>26-UR	ATGTACGAATACGAGATCC<br>TCCACTAGCTCCAGCCAAGGGGCAAGATTGAATGGAC	Amplification of upstream fragment of *FgATG26* for the construction of deletion mutants
26-DF<br>26-DR	AGTAGATGCCGACCGAACATGATTGTTGATTTGGGAATG<br>TTTGTTGGACCGACGAGA	Amplification of downstream fragment of *FgATG26* for the construction of deletion mutants
26-NF<br>26-NR	CGTCCCATCGTGAAGCAG<br>CCATCCTCCTCTGAACCC	Amplification of the final products using for the *FgATG26* gene deletion
26-YF<br>26-YR	CCCCTGAAGAAGTCGCTGAT<br>CGCCCTTGAGTCGAAACC	For identification of *FgATG26* gene deletion transformants
28-UF<br>28-UR	CTGGTCTGGTCCCGTCTG<br>TCCACTAGCTCCAGCCAAGCGGCGTCGAGTGAGAAAA	Amplification of upstream fragment of *FgATG28* for the construction of deletion mutants
28-DF<br>28-DR	AGTAGATGCCGACCGAACACGAACGCCTATGTGAAATCC<br>CAATTGCCTGGTCCAACAC	Amplification of downstream fragment of *FgATG28* for the construction of deletion mutants

28-NF<br>28-NR	GGTAGGCTGATGGCGGAGAT<br>GACACGACCGAAAGACAAGA	Amplification of the final products using for the *FgATG28* gene deletion
28-YF<br>28-YR	AGGTTTGGATCGAATAGCCC<br>TCTCGCACCCACCTACGG	For identification of *FgATG28* gene deletion transformants
29-UF<br>29-UR	TCACTACCACGCCAAACG<br>TCCACTAGCTCCAGCCAAGCTGTCAACAAGAGTCCACCATC	Amplification of upstream fragment of *FgATG29* for the construction of deletion mutants
29-DF<br>29-DR	AGTAGATGCCGACCGAACAGTTGCCATTATCCACCCTG<br>CTTGCTGTGCTGCCGTCC	Amplification of downstream fragment of *FgATG29* for the construction of deletion mutants
29-NF<br>29-NR	CGAAGAGCAAAAGCGTGTAG<br>ACTACCCTTAGCCATACCACC	Amplification of the final products using for the *FgATG29* gene deletion
29-YF<br>29-YR	CACTCCGCTTTATCCTTTCG<br>GGGTTTTCCGTGACTCTTGT	For identification of *FgATG29* gene deletion transformants
33-UF<br>33-UR	CAGTTGGGTTGTCGGATGA<br>TCCACTAGCTCCAGCCAAGTTGTGGCTGTGACCTGTCTG	Amplification of upstream fragment of *FgATG33* for the construction of deletion mutants
33-DF<br>33-DR	AGTAGATGCCGACCGAACAGAGGAATCCTTTCCCTCCC<br>CCCGATAATTCCAACTCCTACC	Amplification of downstream fragment of *FgATG33* for the construction of deletion mutants
33-NF<br>33-NR	GACACCGACTTGTAGGATCAAC<br>TATTCCAAGGGCGACGAA	Amplification of the final products using for the *FgATG33* gene deletion
33-YF<br>33-YR	CACTTGGCTTGCTGACGG<br>CACCCCAAATACCAACGACT	For identification of *FgATG33* gene deletion transformants
HPH-F<br>HPH-R	TATTGAAGGAGCATTTTTGG<br>GCTCTTGTTCGGTCGGCATC	Amplification of *HPH* gene cassette
Neo-F	CCACCGCGGTGGCGGCCGCTCTAGAGGAGGTCAACA CATCAATGCT	Amplification of the *NEO* gene
Neo-R	CCCGGGGGATCCACTAGTTCTAGATCAGAAGAACTC GTCAAGAAG	
GFP-F<br>GFP-R	ATGGTGAGCAAGGGCGAGGA<br>CTTGTACAGCTCGTCCATGC	Amplification of the *GFP* gene cassette

Fg8-F	GCATGGACGAGCTGTACAAGATGCGCAGCAAATTCAAGGA	Amplification of the full length ORF of *FgATG8* gene
Fg8-R	CTAAAGGGAACAAAAGCTGGCGGTGTTCTTCTGCTTT	
Mo1-F	CCCCCGGGCTGCAGGAATTCGGATTTCAAAGATGGGTCGC	Amplification of the upstream fragment (native promoter) of *MoATG1* gene
Mo1-R	GACTCCTGTGGGCCCGCCATATGAGCAAGCACAGTTCAAA	
Fg1-F	ATGGCGGGCCCACAGGAGTC	Amplification of the full length ORF of *FgATG1* gene
Fg1-R	GGTACCGGGCCCCCCCTCGAGTCAGGAAGATGCATGCGAG	
MF1-YF	AGCCCGCCCTATCCTTAC	For identification of complemented transformants of Δ*Moatg1* mutants with *FgATG1* gene
MF1-YR	GGTCCCTGATTTGGTCGTTA	
Mo5-F	CCCCCGGGCTGCAGGAATTCTTGCGTAGTTTGTCTGAGC	Amplification of the upstream fragment (native promoter) of *MoATG5* gene
Mo5-R	CGGGATGGGAGAAGACATAGTTCTGGACCGGCC	
Fg5-F	ATGTCTTCTCCCATCCCGCA	Amplification of the full length ORF of *FgATG5* gene
Fg5-R	GGTACCGGGCCCCCCCTCGAGAAGATTCCAATTGCCAAGCG	
MF5-YF	GGGGTCGCATGGGTGTTT	For identification of complemented transformants of Δ*Moatg5* mutants with *FgATG5* gene
MF5-YR	TCTGCTTGGCGTGTCC	
